# VSIG4^+^ tumor-associated macrophages mediate neutrophil infiltration and impair antigen-specific immunity in aggressive cancers through epigenetic regulation of SPP1

**DOI:** 10.1186/s13046-025-03303-z

**Published:** 2025-02-07

**Authors:** Zongfu Pan, Jinming Chen, Tong Xu, Anqi Cai, Bing Han, Ying Li, Ziwen Fang, Dingyi Yu, Shanshan Wang, Junyu Zhou, Yingying Gong, Yulu Che, Xiaozhou Zou, Lei Cheng, Zhuo Tan, Minghua Ge, Ping Huang

**Affiliations:** 1 Center for Clinical Pharmacy, Cancer Center, Department of Pharmacy, Zhejiang Provincial People’s Hospital (Affiliated People’s Hospital), Hangzhou Medical College, Hangzhou, 310014 China; 2 Otolaryngology & Head and Neck Center, Cancer Center, Department of Head and Neck Surgery, Zhejiang Provincial People’s Hospital (Affiliated People’s Hospital), Hangzhou Medical College, Hangzhou, 310014 China; 3 Zhejiang Key Laboratory of Precision Medicine Research on Head & Neck Cancer, Zhejiang Provincial People’s Hospital, Hangzhou, 310014 China; 4 Zhejiang Provincial Clinical Research Center for Head & Neck Cancer, Zhejiang Provincial People’s Hospital, Hangzhou, 310014 China

**Keywords:** Immunotherapy, Macrophage, Immune checkpoint, VSIG4, Reprogramming

## Abstract

**Abstract:**

V-set and immunoglobulin domain-containing 4 (VSIG4) positive tumor-associated macrophage (VSIG4^+^ TAM) is an immunosuppressive subpopulation newly identified in aggressive cancers. However, the mechanism how VSIG4^+^ TAMs mediate immune evasion in aggressive cancers have not been fully elucidated. In our study, we found targeting VSIG4^+^ TAMs by VSIG4 deficiency or blockade remarkably limited tumor growth and metastasis, especially those derived from anaplastic thyroid cancer (ATC) and pancreatic cancer, two extremely aggressive types. Moreover, the combination of VSIG4 blockade with a BRAF inhibitor synergistically enhanced anti-tumor activity in ATC-tumor bearing mice. VSIG4 deficiency recovered the antigen presentation (B2m, H2-k1, H2-d1) of TAMs and activated antigen-specific CD8^+^ T cells by promoting their in vivo proliferation and intratumoral infiltration. Notably, loss of VSIG4 in TAMs significantly reduced the production of lactate and histone H3 lysine 18 lactylation, resulting the decreased transcription of *SPP1* mediated by STAT3, which collectively disrupted the cell-cell interactions between TAMs and neutrophils. Further combination of VSIG4 with SPP1 blockade synergistically boosted anti-tumor activity. Overall, our studies demonstrate the epigenetic regulation function of VSIG4 confers on TAMs an alternative pattern, beyond the checkpoint role of VSIG4, to shape the immunosuppressive tumor microenvironment and impair antigen-specific immunity against aggressive cancers.

**Graphical abstract:**

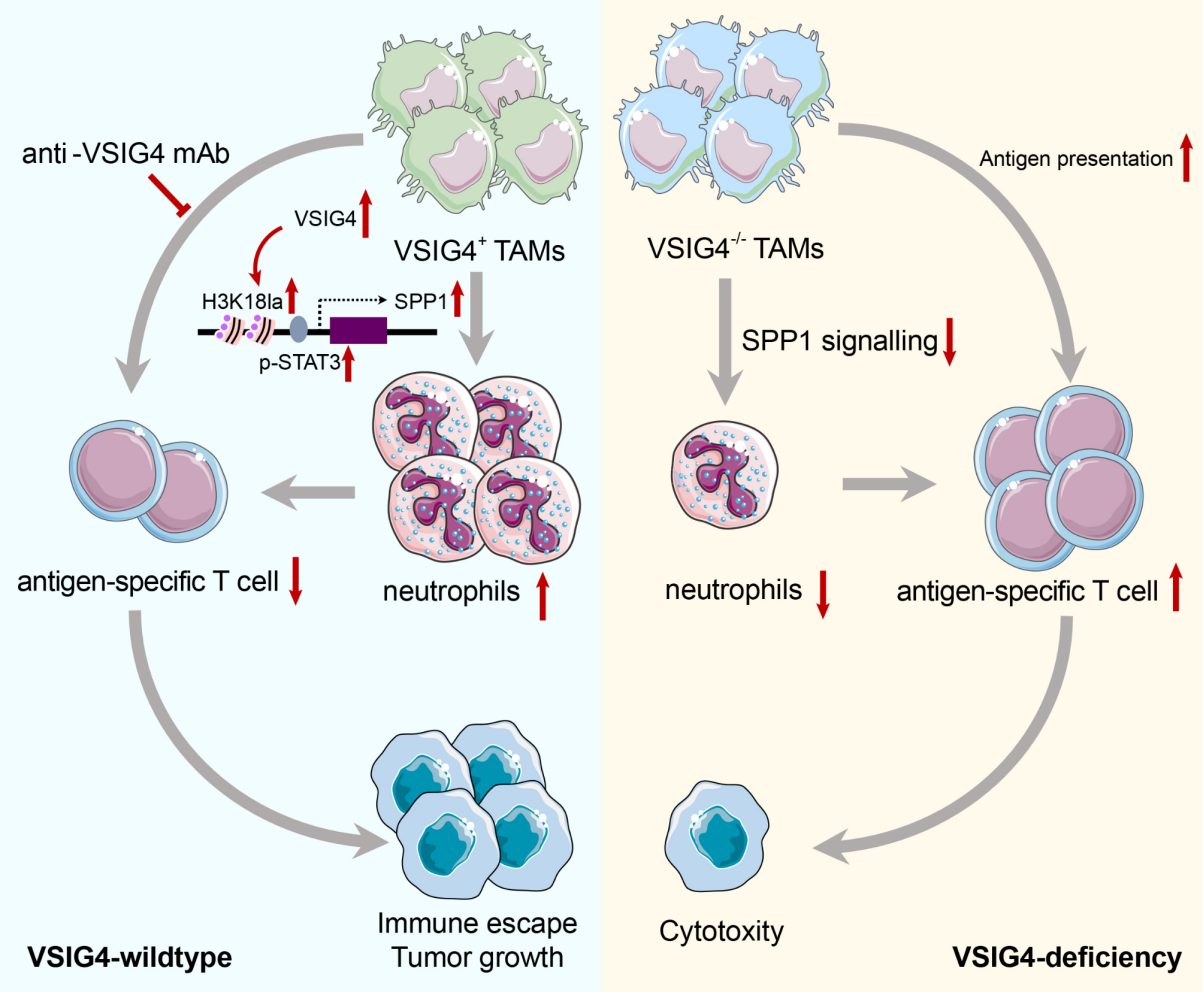

**Supplementary Information:**

The online version contains supplementary material available at 10.1186/s13046-025-03303-z.

## Introduction

Immune checkpoint inhibitors (ICIs), such as PD-1/PD-L1 blockade, have been successfully applied in the clinical treatment of melanoma, non-small cell lung cancer (NSCLC) and other malignant tumors [1, 2]. Atezolizumab treatment demonstrated objective response rate (ORR) ranging from 33.3% in SCLC to higher rate in PD-L1^+^ patients with NSCLC [3, 4]. Patients with microsatellite instability-high or Epstein-Barr virus-positive gastric cancer dramatically benefited from pembrolizumab with ORRs of 85.7% and 100%, respectively [5]. However, targeting PD-1/PD-L1 axis achieved poor outcome in patients with aggressive cancers, owing to the difference in the proportion and functional status of immune cells [6–8]. For example, overall survival (OS) in patients with anaplastic thyroid cancer (ATC) is only 4.8 months, and more than 80% of patients showed no response to anti-PD-1 therapy [9]. The ORR was only 3.1% for metastatic pancreatic ductal adenocarcinoma (PDAC) patients receiving combination therapy of anti-PD-L1 and anti-CTLA4, and 0% for patients receiving monotherapy [10]. Hence, there is an urgent need to search for potential strategies to optimize anti-tumor immunotherapy in those aggressive cancers.

V-set and immunoglobulin domain-containing 4 (VSIG4) is a B7-family protein that predominantly expresses in tissue-resident macrophages, particularly in those of liver, and minimally presents in other cell types, including synovial cells [11, 12]. VSIG4 is known as a receptor of complement iC3b and C3b that inhibits complement activity and negatively regulates the activation of effector T cells [13, 14]. VSIG4 has been implicated in a variety of diseases, particularly those involving immune dysregulation and inflammation, such as non-alcoholic steatohepatitis, rheumatoid arthritis, and obesity-related metabolic disorders [15–17]. VSIG4 can inhibit the activation of proinflammatory macrophages by reprogramming the mitochondrial pyruvate metabolism in inflammatory diseases [18]. VSIG4 was also reported as an independent prognostic factor in multiple myeloma and glioma [19, 20]. Recently, VSIG4^+^ tumor-associated macrophages (TAMs) have been identified as a specific subset that infiltrates aggressive cancers, including anaplastic thyroid cancer (ATC) [14, 21, 22]. VSIG4^+^ TAMs were revealed as a key subpopulation of immunosuppressive microenvironment intensively infiltrating ATC tumors and frequently interacting with immune cells [21]. However, the molecular mechanism by which VSIG4^+^ TAMs shape the immunosuppressive microenvironment have not been fully elucidated.

In this study, the anti-tumor effects by targeting VSIG4^+^ TAMs were investigated using VSIG4-knockout (KO) mouse models and an anti-VSIG4 antibody, especially in two extremely aggressive cancers, PDAC and ATC. The mechanism of VSIG4^+^ TAMs in remodeling the immunosuppressive microenvironment was delineated. Our findings demonstrated that TAM-expressed VSIG4 induced histone H3 lactylation and epigenetically activated *SPP1* transcription, which facilitates neutrophil infiltration and impairs antigen-specific immunity, shedding light on targeting VSIG4^+^ TAMs as a promising strategy for aggressive cancer immunotherapy.

## Materials and methods

### Mouse strains

TPOCreER (#026512), *Braf*^tm1Mmcm/WZ^ (#017837), and *Trp53*^tm1Brn/tm1Brn^ (#008462) mice were purchased from Jackson Laboratory. In this model, three genotypes of mice were crossed to generate the TBP mice. Tamoxifen (Sigma, T5648) was dissolved in corn oil (Sigma, C8267) and administered twice at a dose of 0.1–0.2 mg/g at 8–12 weeks to induce spontaneous formation of ATC [23]. C57BL/6J mice were purchased from Gempharmatech (Nanjing, China). *Vsig4*^*−/−*^ mice were constructed by Cyagen Biosciences (Suzhou, China). The CRISPR/Cas9-mediated genome engineering selectively knocked out the sequence between exons 1 and 2 to generate *Vsig4*^*−/−*^ mice. No obvious pathological phenotypes were observed in *Vsig4*^*−/−*^ mice. OT-I(V1) mice (NM-KI-241328) were purchased from Model Organisms (Shanghai, China). All mice were housed in a specific pathogen-free environment with a 12-h light/dark cycle.

### Cell culture

The cancer cell lines B16F10, E.G7-OVA, and Panc02 were derived from the American Type Culture Collection (ATCC) cell bank. Mouse anaplastic thyroid cancer cell mATC was isolated from primary cells, as reported previously [24]. Briefly, after six months of tamoxifen induction, tumor tissues from TBP mice were isolated and digested using collagenase I to obtain the mATC cell line. E.G7-OVA and mATC cells were cultured in DMEM containing 10% Fetal Bovine Serum (FBS). Panc02 cells were cultured in RPMI-1640 medium supplemented with 10% FBS. All the cells were cultured in a humidified incubator with 5% CO_2_ at 37 °C. B16F10 and Panc02 cells were transfected with ovalbumin (OVA) lentivirus and polybrene (Sirion Biotech, SB-A-LF-900-01) and subsequently screened using puromycin to generate B16F10-OVA and Panc02-OVA cell lines, respectively.

### Isolation of bone marrow-derived macrophages and dendritic cells

6-week-old C57BL/6J mice were sacrificed and bone marrow cells were isolated from the femur. The cell suspension was incubated with the red cell lysate for 5 min and centrifuged at 1000 rpm for 5 min. Subsequently, bone marrow-derived macrophage (BMDM) induction medium containing 20 ng/mL macrophage colony-stimulating factor (M-CSF) (Abclonal, RP01216) or bone marrow-derived dendritic cell (BMDC) induction medium containing 15 ng/mL granulocyte-macrophage colony-stimulating factor (GM-CSF) (Abclonal, RP01206) and 10 ng/mL interleukin-4 (IL-4) (Abclonal, RP01161) were respectively used to culture BMDMs and BMDCs, and fresh medium was replaced on the third day. BMDMs and BMDCs were obtained after seven days of culture.

### Animal models

1 × 10^6^ B16F10, E.G7-OVA, Panc02, and 4 × 10^6^ mATC cells were injected subcutaneously into wild-type and *Vsig4*^*−/−*^ mice (6–8 weeks old, female). Tumor volume was measured every 2 or 3 days and calculated according to the following formula: tumor volume = length × width × width/2.

For the lung metastasis experiment, 100 µL of B16F10-OVA containing 1 × 10^6^ cells were intravenously injected into wild-type or *Vsig4*^*−/−*^ mice. Two weeks later, lung tissue was removed to record the number of lung metastases. Lung tissues were embedded in paraffin to generate 4-µm sections and stained with hematoxylin and eosin (H&E) reagent.

To determine the anti-tumor effect of the VSIG4 antibody on tumor growth, 1 × 10^6^ Panc02 cells were subcutaneously implanted into C57BL/6J mice (6–8 weeks old, female). On day 3, 10 mg/kg VSIG4 antibody (Clone No.8A6E1) was intraperitoneally injected every 3 days. To evaluate the synergistic effect of the VSIG4 blockade and BRAF inhibitor, 4 × 10^6^ mATC cells were injected subcutaneously into C57BL/6J mice. Mice were treated with anti-VSIG4 and/or PLX4720 as a single agent and combination therapy when the mean tumor volume reach 100 mm^3^. 10 mg/kg VSIG4 antibody was intraperitoneal injected every three days, and 30 mg/kg of the BRAF inhibitor PLX4720 (MedMol, S87952) was administered daily. Tumor volume was measured every 3 days.

For the in vivo T cell depletion experiment, 1 × 10^6^ Panc02 cells were injected subcutaneously. Then, mice were intraperitoneally injected with 100 µg anti-CD4 (GK1.5) antibody (Biolegend, 100458), 50 µg anti-NK-1.1 (PK136) antibody (Biolegend, 108760), and 100 µg anti-CD8a (53-6.7) antibody (Biolegend, 100764) on days 1, 3, and 5 according to the groups, respectively. Tumor growth was observed every 2 to 3 days, and the difference in tumor growth in the wild-type group, control in the VSIG4-KO group, aCD4 in the VSIG4-KO group, aCD8 in the VSIG4-KO group, aNK in the VSIG4-KO group, and the combination of aCD8 and aNK in the VSIG4-KO group was investigated.

All animal experiments were conducted in accordance with the Guidelines for the Care and Use of Laboratory Animals (National Institutes of Health, 8th Edition), and complied with ethical guidelines. The experimental protocol was approved by the Animal Ethics Committee of the Zhejiang Provincial People’s Hospital.

### Flow cytometry

The tumor tissue was cut into small pieces and digested with 1 mg/mL collagenase IV (Thermo Fisher, 17104019) in a shaker at 37 °C for 1 h. The cell suspension was then filtered and centrifuged to obtain a single-cell suspension for flow analysis. One hundred microliters of antibody staining solution (dilution ratio: 1:100) was added to fully resuspend the cells, and the samples were incubated at 4 °C in the dark for 30 min. After washing with PBS, the cells were suspended in 200 µL PBS and analyzed by flow cytometry (Beckman Coulter, USA). Intracellular and nuclear protein staining was performed using the BD Cytofix/Cytoperm™ Plus kit or the intracellular staining kit (Biolegend, 421403) according to the manufacturer’s instructions. The antibody information used is summarized in Table S1.

### CD8^+^ T cell sorting and antigen-specific T cell response

Spleens from OT-I mice were collected and pipetted into single cell suspensions. Then, red blood cell lysate (Biolegend, 420302) was added at room temperature for 5 min and centrifuged at 300 × g for 5 min. A CD8^+^ T cell sorting kit (Biolegend, 480035) was used for CD8^+^ T cell sorting under sterile conditions according to the manufacturer’s instructions. 1 × 10^6^ OT-I CD8^+^ T cells were injected intravenously into wild-type and *Vsig4*^*−/−*^ mice on day −1. Mice were immunized on day 0 by intraperitoneal injection of 100 µg of OVA_257 − 264_ peptide (Sigma, 138831-86-4) and 100 µg of polyinosine-polycytidylic acid (poly(I: C)) (MCE, HY-107202). Mice were intraperitoneally administered 5 mg/kg/day 5-Ethynyl-2’-deoxyuridine (EdU) during the experimental period. On day 5, mice were sacrificed, and splenic T cells were stained with anti-CD8 (Biolegend, 100752) and anti-H2Kb-OVA_257 − 264_ (Biolegend, 141608) antibodies for flow cytometry analysis. In addition, the Cell-Light™ Apollo567 staining kit (RiboBio, C10371-1) was used to stain mouse splenic T cells, and the proliferation of OT-I CD8^+^ T cells was analyzed by flow cytometry. To evaluate the tumor infiltration and proliferation of antigen-specific CD8^+^ T cells, 1 × 10^6^ OVA-overexpressing Panc02 cells were subcutaneously implanted into C57BL/6J mice (6–8 weeks old, female). Splenic OT-I CD8^+^ T cells were injected intravenously on day 16, following by immunizing with 100 µg of OVA_257 − 264_ peptide and 100 µg of poly(I: C). 5 mg/kg/day EdU were injected intraperitoneally from day 17, and tumors were harvested for flow cytometry analysis on day 21.

### Gene expression and survival analysis

The expression patterns of VSIG4 and CD274 in human peripheral blood mononuclear cell (PBMC) and 18 tumor tissues were analyzed by the TISCH2 database (http://tisch.comp-genomics.org/home/).

### Single-cell RNA sequencing

Two pairs of mATC-derived tumors from the wild-type and *Vsig4*^−/−^ groups were randomly selected for single-cell RNA sequencing (scRNA-seq) using 10x Genomics. The sequencing data were processed and converted to the FASTQ format using Illumina’s bcl2fastq software (version 2.20).

### Quality control of scRNA-seq data

Raw sequencing data from 10x Genomics were processed using CellRanger (version 6.0.2), with the mouse genome mm10 as the reference [25]. The Seurat R package (version 4.3.1) was used for downstream analysis. Cells with less than 200 unique genes expressed, more than 6,000 unique genes expressed, > 10% mitochondrial reads, or > 0.1% hemoglobin reads were excluded. Doublets were filtered out using “DoubletFinder” R package [26]. Gene expression matrices of the remaining 40,079 cells were normalized. Variable genes were selected using the “FindVariableGenes” function. Data integration was performed using the “IntegrateData” function, followed by scaling with “ScaleData”. Principal component analysis (PCA) identified the top 30 principal components for uniform manifold approximation and projection (UMAP) dimensionality reduction.

### Cell subpopulations clustering analysis and annotation

Cell clustering was conducted with the “FindClusters” function and annotated by canonical marker gene expression. To annotate cell subclusters, differential gene expression (DEGs) analysis was performed to detect significantly overexpressed genes in individual subclusters using the Seurat “FindAllMarkers” function. Each cluster defines markers with a threshold at which gene expression is present in more than 50% of the cells. According to literature and classical marker genes, cell subpopulations were classified as follows: NK cells (*Klrd1*, *Klre1*, *Klrk1*), NKT cells (*Tnfrsf14*, *Klrd1*, *Cd28*), Treg cells (*Foxp3*, *Il2ra*, *Tnfrsf19*), CD8^+^ T cells (*Cd8a*, *Cd8b1*), CD4^+^ T cells (*Cd4*), γδT cells (*Trdc*), mast cells (*Tpsab1*, *Tpsab2*, *Cpa3*), pDC cells (*Siglech*), DC cells (*Flt3*, *Fscn1*), monocyte cells (*Ly6c2*, *Plac8*), macrophage cells (*C1qa*, *C1qb*), neutrophil cells (*Csf3r*), B cells (*Cd79a*, *Jchain*, *Ms4a1*), endothelial cells (*Ramp2*, *Mafb*, *Picalm*), fibroblast cells (Col1a1, *Col1a2*, *Acta2*), tumor cells (*Krt8*, *Krt8*).

T cell subpopulations were classified as follows [8, 27]: naïve CD4^+^ T cells (CD4^+^ T_naïve_ cells; *Lef1*, *Ccr7*, *Tnfrsf14*, *Cd4*), exhausted CD4^+^ T cells (CD4^+^ T_EX_ cells; *Hif1a*, *Lag3*, *Ctla4*, *Cd4*), effect memory CD8^+^ T cells (CD8^+^ T_EM_ cells; *Gzmk*, *Gzmb*, *Nkg7*, *Cd8a*), central memory CD8^+^ T cells (CD8^+^ T_CM_ cells; *Anxa1*, *Gzmk*, *Lag3*, *Cd8a*), naive CD8^+^ T cells (CD8^+^ T_naïve_ cells; *Tcf7*, *Sell*, *Lef1*, *Cd8a*), proliferation CD8^+^ T cells (CD8^+^ T_prolieration_ cells; *Stmn1*, *Mki67*, *Top2a*, *Cd8a*), Treg cells (*Foxp3*, *Il2ra*, *Tnfrsf19*), γδT cells (*Trdc*).

DEGs between macrophages from wild-type and *Vsig4*^−/−^ groups were identified using the FindMarkers function from the Seurat package. The ggplot2 package was used to generate violin plots, and the Ggradar package was used to create radar charts. Differences between sample groups were assessed using the Wilcoxon test, with a *P* value < 0.05 considered statistically significant.

### Metascape analysis

The top 100 upregulated DEGs of macrophages from the wild-type and *Vsig4*^−/−^ groups were used to perform Metascape analysis (https://metascape.org/gp/index.html#/main/step1) for annotation of biological functions. Databases used in the Metascape pathway enrichment analysis including Gene Ontology (GO), Kyoto Encyclopedia of Genes and Genomes (KEGG), Reactome, and MSigDB.

### Custom gene set enrichment analysis (GSEA)

Custom gene sets, including positive regulation of mononuclear cell migration (*Csf1*, *Lgals3*, *Cxcl14*, *Pycard*), neutrophil chemotaxis (*Lgals3*, *Ccl6*, *Spp1*, *Jaml*), regulation of mononuclear cell proliferation (*Arg1*, *Csf1*, *Il1a*, *Lgals3*, *Anxa1*, *Mif*, *Pycard*), negative regulation of leukocyte activation (*Arg1*, *Fn1*, *Ldlr*, *Lgals3*, *Anxa1*), and regulation of T cell proliferation (*Arg1*, *Il1a*, *Lgals3*, *Anxa1*, *Pycard*), were obtained from Metascape analysis. GSEA analysis was conducted using these custom gene sets with parameter sets to identify significant enrichment. The results were visualized using a radar chart, providing insights into the enrichment score of defined biological processes across *SPP1*^+^*VSIG4*^+^ and *SPP1*^−^*VSIG4*^−^ macrophage clusters. Macrophage cells were obtained from ATC and normal samples in the GSE148673 and GSE193581 datasets. The cells were classified into four subgroups based on their expression levels of *VSIG4* and *SPP1*. The median expression levels of *VSIG4* and *SPP1* were calculated. A function was then applied to categorize each cell: cells with *VSIG4* expression above and *SPP1* above their respective medians were classified as “*SPP1*^+^*VSIG4*^+^ macrophage”, *VSIG4* and *SPP1* below as “*SPP1*^−^*VSIG4*^−^ macrophage”.

### Cell-cell communication analysis

Cellchat was used to analyze cell–cell communications [28]. CellChat quantifies ligand-receptor interactions by mapping gene expression data onto a curated database of known interactions. The results were visualized to reveal cell-cell communication networks and the roles of specific signaling pathways in mediating interactions between different cell types from wild-type and *Vsig4*^−/−^ groups, simultaneously comparing the types, quantities, and strengths of communications.

### RT-qPCR and western blot

The induced BMDMs isolated from wild-type or *Vsig4*^*−/−*^ mice were seeded in a 6-well plate and co-cultured with 5 × 10^4^ mATC cells in an upper chamber of 0.4-µm thickness for 48 h. To evaluated the effects of Stat3 inhibition or lactate on Spp1 expression, BMDMs were treated with 10 µM Stat3 inhibitor Stattic (selleck, 19983-44-9) or 30 mM sodium L-Lactate (Absin, abs42027500) for 48 h. BMDMs were collected to extract mRNA and reversely transcribed according to the instructions of the RNA extraction kit (ES science, RN001), and SYBR Green qPCR Mix (ES science, QP002) was used for fluorescence quantification. The primers were listed in Table S2. For western blotting, proteins were separated by electrophoresis using 10% SDS-PAGE and transferred to a PVDF membrane (Millipore, IPFL00010). Samples were blocked by 5% skim milk at room temperature for 1 h. Primary antibodies against Spp1 (Affinity, AF0227), Stat1 (Proteintech, 10144-2-AP), Stat3 (Proteintech, 10253-2-AP), p-Stat1 (Proteintech, HKP0249), p-Stat3 (Proteintech, HKP0250), p-Stat5 (Proteintech, YP0253), p-Stat6 (Proteintech, YP0256), L-Lactyl-Histone H3 (Lys18) (PTMBio, PTM-1427RM) were incubated at 4 ℃. The corresponding secondary antibodies (Dawen Blotec, China) were incubated at room temperature for 1 h. ECL (MeilunBio, MA0186-1) (liquid A: liquid B = 1:1) was used for chemiluminescence detection, and images were captured by gel imager system.

### Analysis of histone H3 lactylation

The dataset GSE192358 was retrieved from GEO database to explore the accumulation of histone H3 lactylation on *Spp1*. Comparison analysis of histone H3 lysine 18 lactylation (H3K18la) peaks between control and lactate treatment were conducted in IGV2.17.4 software. The online tool Eukaryotic Promoter Database (https://epd.expasy.org/epd) was used to predict the transcription start site and promoter of *Spp1*.

### Dual luciferase reporter gene assay system

The promoter sequence of *SPP1* with 2000 bp upstream of TSS was constructed into the pGL3-basic plasmid, and the target binding sequence (tttttcagaaa) of STAT3 on SPP1 promotor was replaced with gggggggcgct to generate mutant type. The plasmid pRL-TK was set as reference. STAT3 was overexpressed using vector pcDNA3.1. Before transfection, pRL-TK was sequentially mixed with pcDNA3.1 and pGL3-basic to ensure a total amount of 2 µg in 200 µL jetPRIME^®^ buffer (Polyplus, France). Then, 4 µL jetPRIME^®^ was added, mixed, and incubated at room temperature for 10 min. 293T cells were transfected with 200 µL transfection mixture. After 48 h, the dual luciferase reporter gene detection kit (Beyotime, China) was used for detection. The firefly luciferase and renilla luciferase were detected in turn, and the renilla luciferase was used as the internal reference to evaluate the activity of STAT3 on the target reporter gene between different samples according to the obtained ratio.

### Detection of intracellular lactate

To evaluate the lactate in macrophage in vitro, BMDM cells from the bone marrow of WT mice and knockout VISG4 mice were isolated and induced as described above, followed by co-culture with mATC cells for 2 days. To determine the lactate production in TAMs, we isolated TAMs from tumors utilizing the MojoSort™ Mouse F4/80 Selection Kit (480170, BioLegend). The cells obtained from the digested tumors were resuspended in 300 µL of MojoSort buffer and transferred to 5 mL tubes. Subsequently, cells were mixed thoroughly after adding 10 µL of anti-mouse F4/80 biotin-antibody cocktail and incubated on ice for 15 min. 10 µL of Streptavidin Nanobeads were added and incubated on ice for 15 min. 2.5 mL of MojoSort buffer was then added to the tube and placed on a magnetic stand for 5 min. TAM cells were purified after washing by MojoSort buffer for three times. BMDM cells and TAMs were collected to detect the intracellular lactate using lactic acid content detection kit (Abbkine, China). The standard curve was set according to the instructions to calculate the lactate concentration. Subsequently, the protein concentration was detected by the protein quantification kit (Abbkine, China), and the lactate content was normalized according to the protein concentration.

### Statistical analysis

For animal experiments, data are described as the mean ± standard error of the mean (S.E.M.). In vitro results are shown as mean ± standard deviation (S.D.). Significant differences between two or multiple groups were analyzed using two-tailed Student’s t-test and one-way analysis of variance (ANOVA), respectively. Asterisks denote statistical significance (*P < 0.05, **P < 0.01, ***P < 0.001).

## Results

### Targeting VSIG4^+^ TAMs limited tumor growth and metastasis in aggressive cancers

**Fig. 1:**
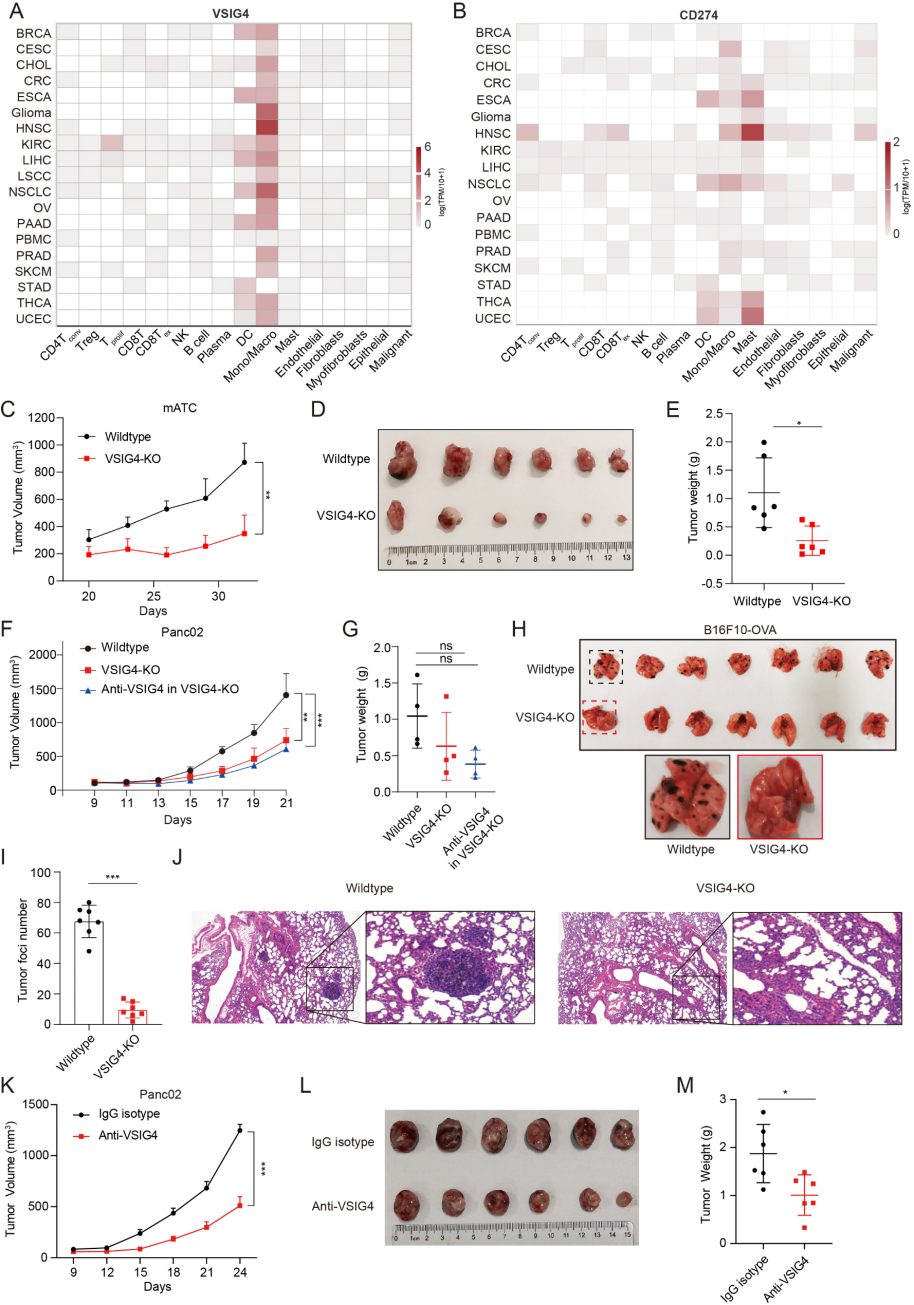
Inhibition of VSIG4^+^TAMs limited tumor growth in aggressive caner models. (**A**-**B**) The expression of VSIG4 and CD274 in different tumor tissues was analyzed by single-cell transcriptome in TISCH2 database. (**C**-**E**) Wild-type (co-housed C57BL/6) (*n* = 6) and VSIG4-KO mice (*n* = 6) were subcutaneously implanted with 4 × 10^6^ mATC. The tumor growth curve was recorded. The tumor size and weight on day 32 were shown. (**F**-**G**) The tumor growth curve and weight (on day 21) of control, VSIG4-KO or VSIG4-KO combined with anti-VSIG4 group in pancreatic tumor model (*n* = 4 for each group). Wild-type and VSIG4-KO mice were subcutaneously implanted with 1 × 10^6^ Panc02. 10 mg/kg VSIG4 antibody was intraperitoneally injected every 3 days. (**H**-**J**) The lung metastatic foci of tumor and H&E staining of lung tissues on day 14. Wild-type (*n* = 7) and VSIG4-KO mice (*n* = 7) were challenged intravenously with 1 × 10^6^ B16F10-OVA to construct lung metastasis model. (**K**-**M**) The tumor growth curve, size and weight of control group and anti-VSIG4 group in pancreatic cancer models subcutaneously implanted with 1 × 10^6^ Panc02. Tumors were collected on day 24, *n* = 6 for each group. 10 mg/kg VSIG4 antibody was intraperitoneally injected every 3 days. Data are a representative of at least two separate experiments. Data are presented as mean ± S.E.M. *P < 0.05, **P < 0.01, ***P < 0.001

The expression of VSIG4 in different cell lineages was firstly evaluated in 18 cancers by single-cell transcriptome. Pan-cancer analysis showed that VSIG4 was strongly expressed in TAMs and dendritic cells (DCs), and a low abundance of VSIG4 was found in malignant cells and lymphocytes. Of note, we found that macrophages and DCs from cancer patients expressed a higher amount of VSIG4 than their counterparts from peripheral blood mononuclear cells of healthy donors (Fig. 1A). Macrophages and DCs derived from the bone marrow of wild-type mice showed low proportion of VSIG4 expression, while flow cytometry analysis of pancreatic cancer cell line Panc02-derived tumors supported that the proportion and mean fluorescent intensity of VSIG4 were highest in TAMs compared to DCs, MDSCs and tumor cells (Fig. S1A-B). CD274 (encoding PD-L1) was expressed diffusely in subpopulations of cancers, and its abundance was weaker than that of VSIG4. In addition, the amount of CD274 showed no obvious increase in some cancers when compared with counterparts from healthy donors, especially those in PDAC, breast cancer, and ovarian cancer (Fig. 1B), indicating VSIG4^+^ TAMs is an unignorable subpopulation in the TME.

To evaluate the role of VSIG4^+^ TAMs in tumor growth and progression, we selected highly aggressive PDAC and ATC as main research subjects. Cancer cell lines were inoculated into VSIG4-deficient (KO) and wild-type (WT) mice. Deletion of VSIG4 remarkably reduced the growth of mATC-derived tumors to approximately 35% compared to wild-type tumors (Fig. 1C-E). Similar effects were observed in VSIG4-KO mice bearing Panc02-, B16F10-, and E.G7-OVA-derived tumors (Fig. 1F-G, S2A-E). Additional treatment with anti-VSIG4 antibody in VSIG4-KO mice slightly improved the anti-tumor effect compared with untreated VSIG4-KO mice (Fig. 1F-G, S2A-E), indicating that VSIG4 expression by the host immune cells, not the tumor cells, was the major factor mediating immune evasion. In addition, melanoma cells were intravenously injected into syngeneic mice to promote the formation of lung metastases. Given that B16F10 cells are characterized by a low ability to induce CD8^+^ T cell responses, B16F10 cells stably expressing the OVA antigen were constructed. The results showed that VSIG4-KO mice exhibited only 1/6 of the metastatic foci found in WT mice (Fig. 1H-J).

We then evaluated the therapeutic effect of VSIG4 blockade in PDAC and ATC. Anti-VSIG4 treatment significantly inhibited tumor growth and reduced the tumor weight in pancreatic cancer (Fig. 1K-M). *BRAF*^*V600E*^ inhibitors have become a standard treatment for *BRAF*^*V600E*^-mutant cancers, including melanoma [29]. Although the initial responses are impressive, the durability of anti-tumor activity is limited owing to the development of resistance [30, 31]. Therefore, we attempted to evaluate the combined treatment of VSIG4 antibody and BRAF inhibitor in ATC, a malignancy with a high frequency of *BRAF*^*V600E*^ mutations. Notably, treatment with anti-VSIG4 antibody or PLX4720 alone exhibited anti-tumor effects, and combination therapy with VSIG4 blockade and *BRAF*^*V600E*^ inhibitor synergistically enhanced anti-tumor activity (Fig. S2F-H). Taken together, these results suggested the pivotal roles of VSIG4^+^ TAMs in the growth and progression of aggressive tumors.

### Targeting VSIG4^+^ TAMs reversed tumor immunosuppressive microenvironment

**Fig. 2:**
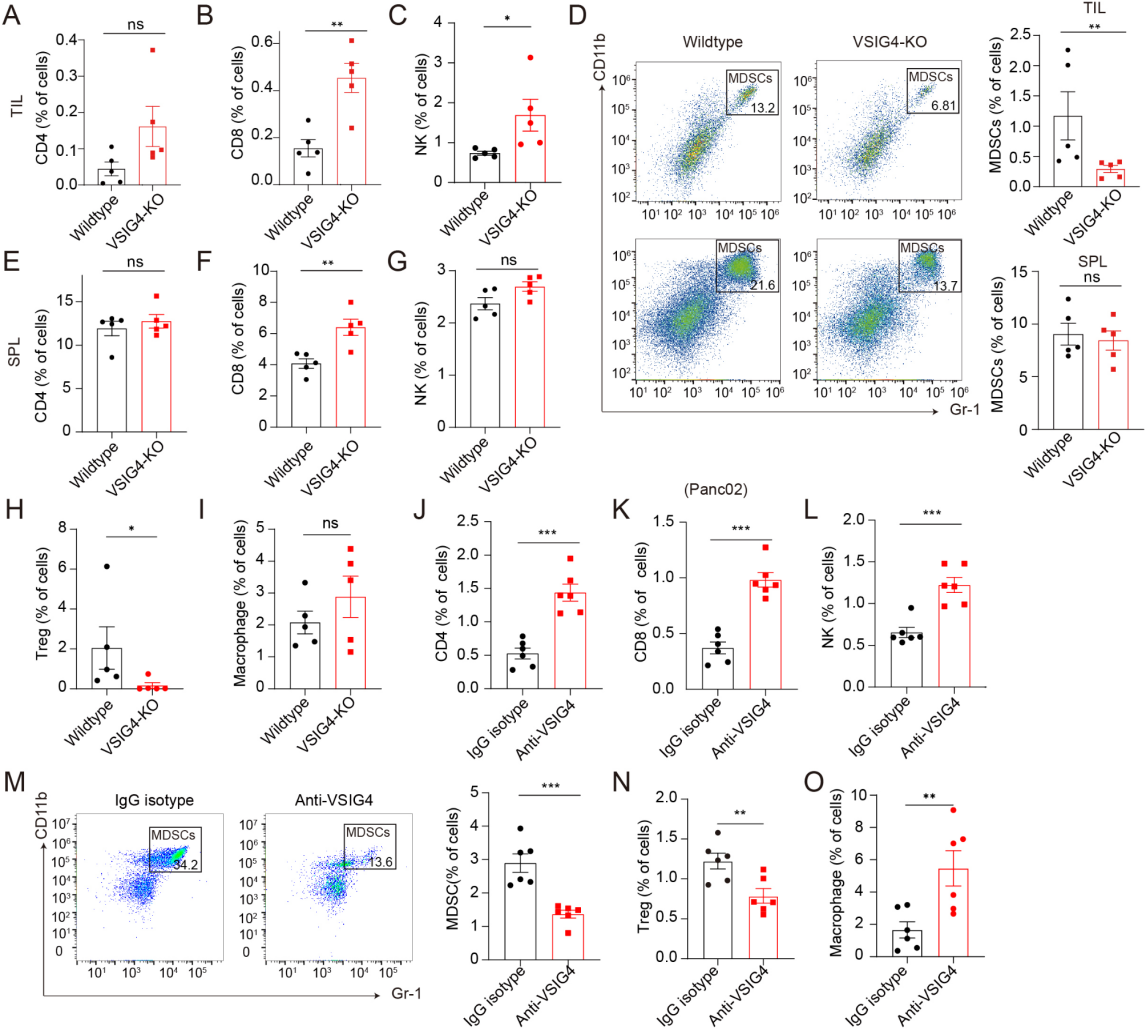
Targeting VSIG4 remodeling suppressive tumor microenvironment. (**A**-**C**) The proportions of tumor-infiltrating NK, CD4^+^ and CD8^+^ T cells were measured by flow cytometry in wild-type (*n* = 5) and VSIG4-KO (*n* = 5) mice bearing E.G7-derived tumor. (**D**) Representative plots and summary histograms showing proportion of MDSCs (CD11b^+^ Gr1^+^) in the tumors of E.G7-bearing wild-type and VSIG4-KO mice (*n* = 5 for each group). (**E**-**G**) The splenic proportions of lymphocytes in lymphoma-bearing mice were examined by flow cytometry in control and VSIG4-KO groups (*n* = 5 for each group). (**H**-**I**) The proportions of tumor-infiltrating Tregs (CD4^+^ CD25^+^ Foxp3^+^) and TAMs (CD11b^+^ F4/80^+^) in wild-type (*n* = 5) and VSIG4-KO (*n* = 5) mice bearing E.G7-derived tumor. (**J**-**O**) The proportions of tumor-infiltrating lymphocytes (TILs) in pancreatic tumor-bearing mice measured by flow cytometry in IgG isotype (*n* = 6) and anti-VSIG4 groups (*n* = 6). Data are presented as mean ± S.E.M. *P < 0.05, **P < 0.01, ***P < 0.001

Furthermore, we analyzed the effects of VSIG4^+^ TAMs intervention on the tumor microenvironment by flow cytometry (Fig. S3 A). Compared with the control group, intratumor infiltration of CD4^+^ T cells and TAMs in mouse lymphoma tumors showed an increasing trend. CD8^+^ T and NK cells were significantly upregulated in the VSIG4-KO group, while MDSCs and Tregs in the VSIG4-KO group were significantly downregulated. In splenic immune cells, CD8^+^ T cells in the VSIG4-KO group were significantly upregulated compared with those in the control group, while other immune cells showed no significant difference (Fig. 2A-I). Subsequently, we examined the effect of anti-VSIG4 therapy on the proportion of immune cells in the pancreatic tumor models. The proportion of CD4^+^ T, CD8^+^ T, NK, and TAMs was significantly upregulated in the anti-VSIG4 group compared to that in the control group, and MDSCs as well as Tregs were evidently downregulated after anti-VSIG4 treatment (Fig. 2J-O). Treatment with anti-VSIG4 antibody also increased the number of CD8^+^ T cells in the spleen compared to that in the control group, while CD4^+^ T cells were not significantly changed (Fig. S3 B-C). We also verified the effects of VSIG4 blockade on tumor-infiltrating lymphocytes (TILs) in the mATC tumor model and obtained similar results (Fig. S3 D-H). In summary, targeting VSIG4^+^ TAMs effectively enhanced anti-tumor immunity by increasing the number of cytotoxic lymphocytes.

### Anti-tumor immunity of VSIG4 inhibition depended on CD8^+^ T cells

**Fig. 3:**
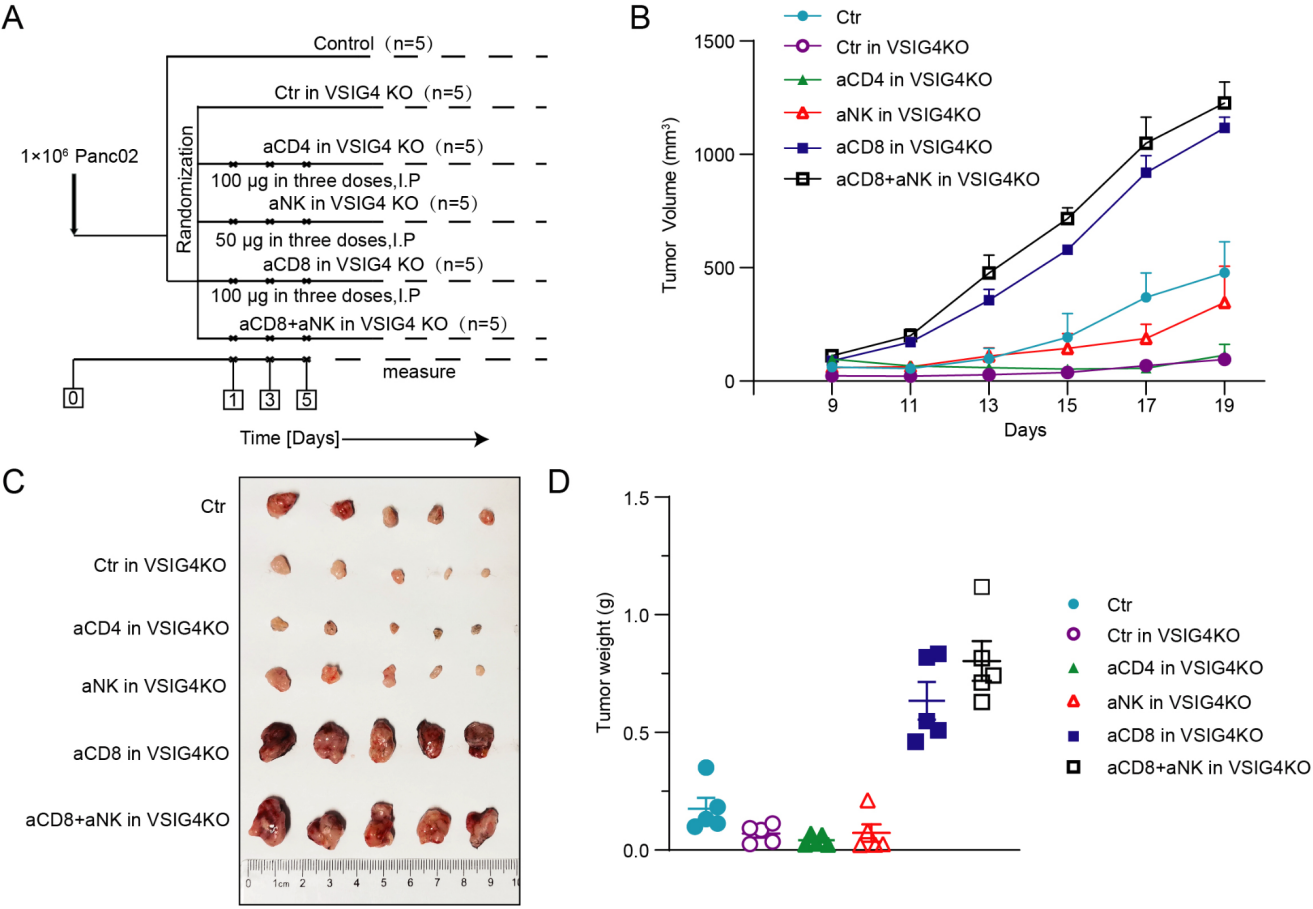
The anti-tumor effect of VSIG4^+^ TAMs inhibition is depended on CD8 ^**+**^ T cells. (**A**) Scheme of animal experiments to evaluate the effects of lymphocyte depletion on tumor growth in VSIG4-KO mice. 1 × 10^6^ Panc02 cells were injected subcutaneously on day 0, and 100 µg CD4 depletion antibody, 100 µg CD8 depletion antibody, 50 µg NK depletion antibody were administered on day 1, 3 and 5. The tumor growth curve (**B**), tumor size (**C**) and tumor weight (**D**) of each group were recorded. Tumors were isolated on day 19, and *n* = 5 for each group. Data are presented as mean ± S.E.M

To understand the mechanism of VSIG4 in tumor development, the potential functional target cells of VSIG4, including CD4^+^, CD8^+^, and NK cells, were depleted with specific monoclonal antibodies after tumor harboring (Fig. 3A). Intriguingly, deletion of CD8^+^ T cells significantly repaired the tumor growth restriction caused by VSIG4-KO, while deletion of CD4^+^ T cells did not affect tumor growth in the VSIG4-KO group (Fig. 3B-D). We also confirmed that the depletion of NK cells reduced the anti-tumor effect of VSIG4-KO to a certain extent and that the double depletion of CD8^+^ T and NK cells showed a slight synergistic effect. Overall, these results indicated that the anti-tumor immunity induced by VSIG4 inhibition was primarily depended on CD8^+^ T cells.

### Targeting VSIG4^+^ TAMs enhanced antigen-specific CD8^+^ T cell function

**Fig. 4:**
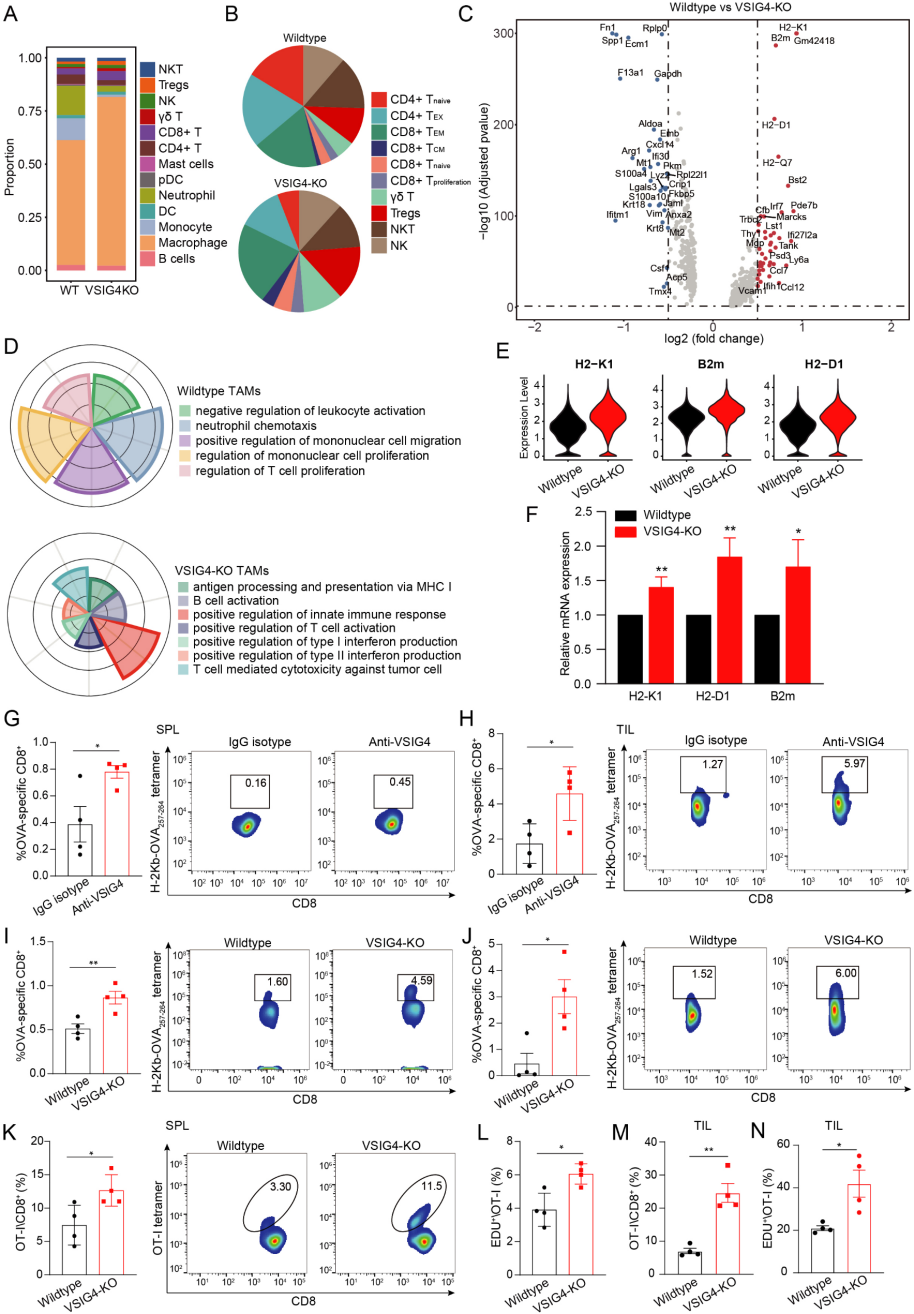
Targeting VSIG4 activated antigen-specific CD8^+^ T cells. (**A**) The single-cell transcriptome analysis of the composition of microenvironmental cells. Samples were isolated from mATC-derived tumors in VSIG4-KO and WT mice on day 32. (**B**) The effect of VSIG4 knockout on tumor infiltration of T lymphocyte subtypes. (**C**) Differentially expressed genes (DEGs) in tumor-associated macrophages (TAMs) after VSIG4 knockout were identified by single-cell RNA sequencing. (**D**) Gene ontology annotation of DEGs in TAMs of VSIG4-KO and WT groups. (**E**) The expressions of H2-K1, B2m, and H2-D1 after VSIG4 knockout in TAMs. (**F**) Co-culture of mATC with BMDM isolated from VSIG4-KO and WT mice to examine the expressions of H2-K1, B2m, and H2-D1 (*n* = 3 for each group). (**G**-**H**) The splenic and intratumor proportions of OVA-specific CD8^+^ T in E.G7 tumor-bearing mice after anti-VSIG4 antibody treatment (*n* = 4 for each group). (**I**-**J**) The splenic and intratumor proportions of OVA-specific CD8^+^ T in WT and VSIG4-KO mice bearing E.G7-derived tumor (*n* = 4 for each group). (**K**-**L**) 1 × 10^6^ splenic CD8^+^ T cells from OT-I(V1) mice were transfused intravenously into VSIG4-KO and WT mice on day −1, following by intraperitoneal injection of 100 µg OVA_257 − 264_ peptide plus 100 µg poly(I: C) on day 0. Splenic OT-I CD8^+^ T cells were examined by H-2Kb-OVA_257 − 264_ tetramer (OT-I tetramer) and anti-CD8 antibody using flow cytometry on day 5 (*n* = 4 for each group). Mice were intraperitoneally injected 5 mg/kg/day EdU from day 0 to detect the percentage of EdU^+^ OT-I T cells (*n* = 4 for each group). (**M**-**N**) Tumor-infiltrating OT-I CD8^+^ T cells and EdU^+^ OT-I T cells were detected in VSIG4-KO and WT mice bearing Panc02-OVA-derived tumor (*n* = 4 for each group). Splenic OT-I CD8^+^ T cells were injected intravenously on day 16, following by intraperitoneal injection of 5 mg/kg/day EdU from day 17, and tumors were isolated on day 21. Data are presented as mean ± S.E.M. *P < 0.05, **P < 0.01

In view of the key regulatory properties of VSIG4^+^ TAMs in tumor immunity, the specific mechanism by which VSIG4^+^ TAMs promote tumor immune escape was explored by single-cell transcriptome analysis of mATC-derived tumors. After descending and unsupervised cell clustering, cells were classified into 16 types based on classical marker genes (Fig. S4 A). The proportion of immune cells showed that CD8^+^ T, γδT, macrophages, and Treg cells increased in the VSIG4-KO group, while CD4^+^ T cells, neutrophils, and monocytes evidently decreased (Fig. 4A). In-depth infiltration analysis of T cell subtypes indicated that cytotoxic cells, including central memory CD8^+^ T, proliferation CD8^+^ T, and γδ T, dramatically increased in the VSIG4-KO group, while naïve CD4^+^ T and exhausted CD4^+^ T cells were remarkably decreased in the VSIG4-KO group (Fig. 4B, S4 B-C). Myeloid cells are the major components of tumor-infiltrating immune cells, and their changes were analyzed. The proportions of neutrophils, monocytes, and mast cells in the transplanted tumor tissues of VSIG4-KO mice were profoundly reduced compared to those of wild-type mice (Fig. S4 D-F). The proportion of neutrophils expressing Ly6g and Wfdc17 decreased from 16.1 to 3.21% (Fig. S4 E-F).

Next, we compared gene expression changes in TAMs in mATC-derived tumors (Fig. 4C, S4 G). The differentially expressed genes (DEGs) of TAMs in the wild-type group were mainly enriched in pro-tumor pathways, including neutrophil chemotaxis, positive regulation of mononuclear cell migration, and regulation of mononuclear cell proliferation. The DEGs including H2-K1, B2m, and H2-D1 in VSIG4-KO TAMs were significantly upregulated and related with anti-tumor signals containing positive regulation of the innate immune response, T cell-mediated cytotoxicity against tumor cells, and antigen-presenting and presentation via MHC I (Fig. 4D-E). In vitro co-culture assay confirmed that knockout of VSIG4 in BMDMs significantly enhanced the expression of H2-K1, B2m, and H2-D1 (Fig. 4F), indicating a negative role of VSIG4 in regulating antigen presentation ability of TAMs.

Notably, VSIG4 knockout enhanced the anti-tumor immunity of macrophages by increasing antigen-presentation associated ligand-receptor pairs between macrophages and CD8^+^ T cells (Fig. S5 A). In addition, we also found that CCL6-CCR1/2 and CSF signaling, which are fundamental for myeloid recruitment and survival, were sharply reduced in the VSIG4-KO group (Fig. S5 A-B). We then explored the effects of VSIG4^+^ TAMs inhibition on antigen-specific CD8^+^ T cell responses. Given that E.G7 expresses the OVA antigen, antigen-specific CD8^+^ T cells isolated from E.G7 tumor-bearing mice were examined. The ratio of OVA-reactive CD8^+^ T cells in the spleen increased significantly in VSIG4-KO and VSIG4 antibody-treated mice compared to that in the control group, and similar results were also found in tumor tissues (Fig. 4G-J). Subsequently, the influence of VSIG4 inhibition on antigen-specific CD8^+^ T cell responses was investigated in vivo using the OT-I mouse model. We extracted splenic CD8^+^ T cells from OT-I mice stimulated with ovalbumin and transfected them into VSIG4-KO mice to determine the proportion and proliferation of OT-I/CD8^+^ T cells. The proportion of OT-I CD8^+^ T cells was significantly higher in the VSIG4-deficient mice than that in the WT mice (Fig. 4K). Consistent with this finding, a higher proliferation ability of OT-I CD8^+^ T cells was observed in VSIG4-KO mice, as indicated by the incorporation of EdU (Fig. 4L). Notably, TAMs in VSIG4-KO mice bearing Panc02-OVA-derived tumors effectively induced intratumor infiltration of OT-I CD8^+^ T cells and promote antigen-dependent proliferation of CD8^+^ T cells compared to those in wild-type mice (Fig. 4M-N). These results suggest that targeting VSIG4^+^ TAMs enhanced the activation of antigen-specific CD8^+^ T cells.

### VSIG4^+^ TAMs recruited neutrophil to shape the immunosuppressive microenvironment by regulating SPP1 signaling

**Fig. 5:**
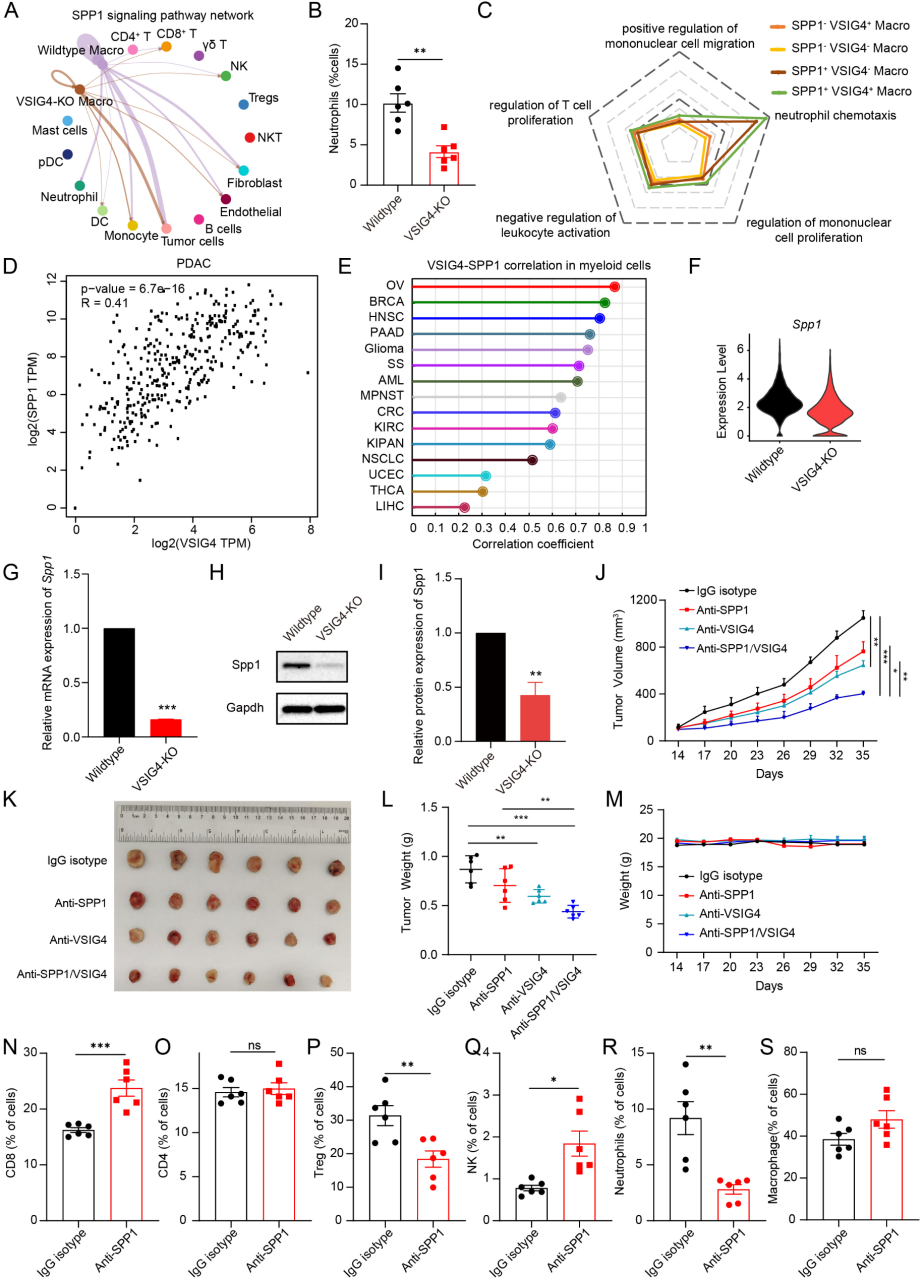
VSIG4^+^ TAMs regulating SPP1 signaling to recruit neutrophil and shaped the immunosuppressive microenvironment. (**A**) The differences of SPP1 signaling in cell-cell communication after VSIG4 knockout. (**B**) The proportions of tumor-infiltrating neutrophils (CD11b^+^ Ly6G^+^) were measured by flow cytometry in wild-type (*n* = 6) and VSIG4-KO (*n* = 6) mice bearing mATC-derived tumor. (**C**) Gene set enrichment analysis to evaluate the scores of indicated pathways in different TAM subpopulations in human ATC samples. (**D**) The correlation between VSIG4 and SPP1 in PDAC was retrieved from GEPIA database. (**E**) Pan-cancer analysis of the correlation between VSIG4 and SPP1 in myeloid cells using TISCH2 database. (**F**) The expressions of Spp1 after VSIG4 knockout in TAMs. (**G**-**I**) Co-culture of mATC with BMDM isolated from VSIG4-KO and WT mice to examine the expressions of Spp1 by RT-qPCR and western blotting (*n* = 3 for each group). Data are presented as mean ± S.D. (**J**-**M**) The mATC tumor-bearing mice were intraperitoneally treated with 10 mg/kg anti-VSIG4 and/or anti-SPP1 as single agent and combination therapy every 3 days, respectively. Tumors were isolated on day 35. The tumor growth curve, body weight, and tumor weight of each group were recorded. (**N**-**S**) The proportions of TILs in ATC tumor-bearing mice measured by flow cytometry in IgG isotype (*n* = 6) and anti-SPP1 groups (*n* = 6). Data are presented as mean ± S.E.M. *P < 0.05, **P < 0.01, ***P < 0.001

Cell-cell interaction analysis revealed a high frequency of interaction between TAMs and other cells through SPP1 signaling in wild-type mice. This communication was reduced in VSIG4-KO mice (Fig. 5A, S5 B). Specifically, SPP1 signaling-mediated communication between TAMs and neutrophils was disrupted in the VSIG4-KO group. Compared with wild-type mice, flow cytometry analysis supported that the proportion of neutrophils was significantly reduced in VSIG4-KO mice bearing mATC-derived tumors (Fig. 5B). We then validated the function of SPP1^+^ VSIG4^+^ TAMs in human ATC tumors. Interestingly, SPP1^+^ VSIG4^+^ TAMs exhibited stronger activity of neutrophil chemotaxis than SPP1^−^ VSIG4^−^ TAMs. In addition, we noticed that SPP1^+^ VSIG4^−^ TAMs also show strong activity of neutrophil chemotaxis, suggesting that SPP1 signaling is essential for shaping the immunosuppressive microenvironment (Fig. 5C). The expression of VSIG4 showed high correlation with SPP1 in PDAC tissues (Fig. 5D). Moreover, pan-cancer analysis also demonstrated a high correlation between VSIG4 and SPP1 in myeloid cells (Fig. 5E). Compared to wild-type mice, TAMs in VSIG4-deficiency mice showed significant downregulation Spp1 (Fig. 5F). Similar results by co-culture assay confirmed that knockout of VSIG4 in BMDMs significantly reduced the expression of Spp1 (Fig. 5G-I). Considering SPP1^+^ VSIG4^−^ TAMs also possessed strong activity of neutrophil chemotaxis, we then explored whether simultaneously inhibiting VSIG4 and SPP1 could improve the anti-tumor effect against ATC. We found mice treated with anti-VSIG4 or anti-SPP1 antibody alone exhibited slower tumor growth, and combination therapy with VSIG4 and SPP1 blockade synergistically enhanced anti-tumor activity without affecting body weight (Fig. 5J-M). Analysis of TILs revealed that SPP1 blockade significantly enhanced the proportion of CD8 and NK cells, while neutrophils and Tregs were evidently decreased (Fig. 5N-S). Collectively, VSIG4^+^ TAMs reshaped the immune microenvironment by facilitating SPP1-mediated neutrophils recruitment and impairing antigen presentation.

### VSIG4 facilitated histone H3 lactylation by increasing lactate production and epigenetically activated *SPP1* transcription

**Fig. 6:**
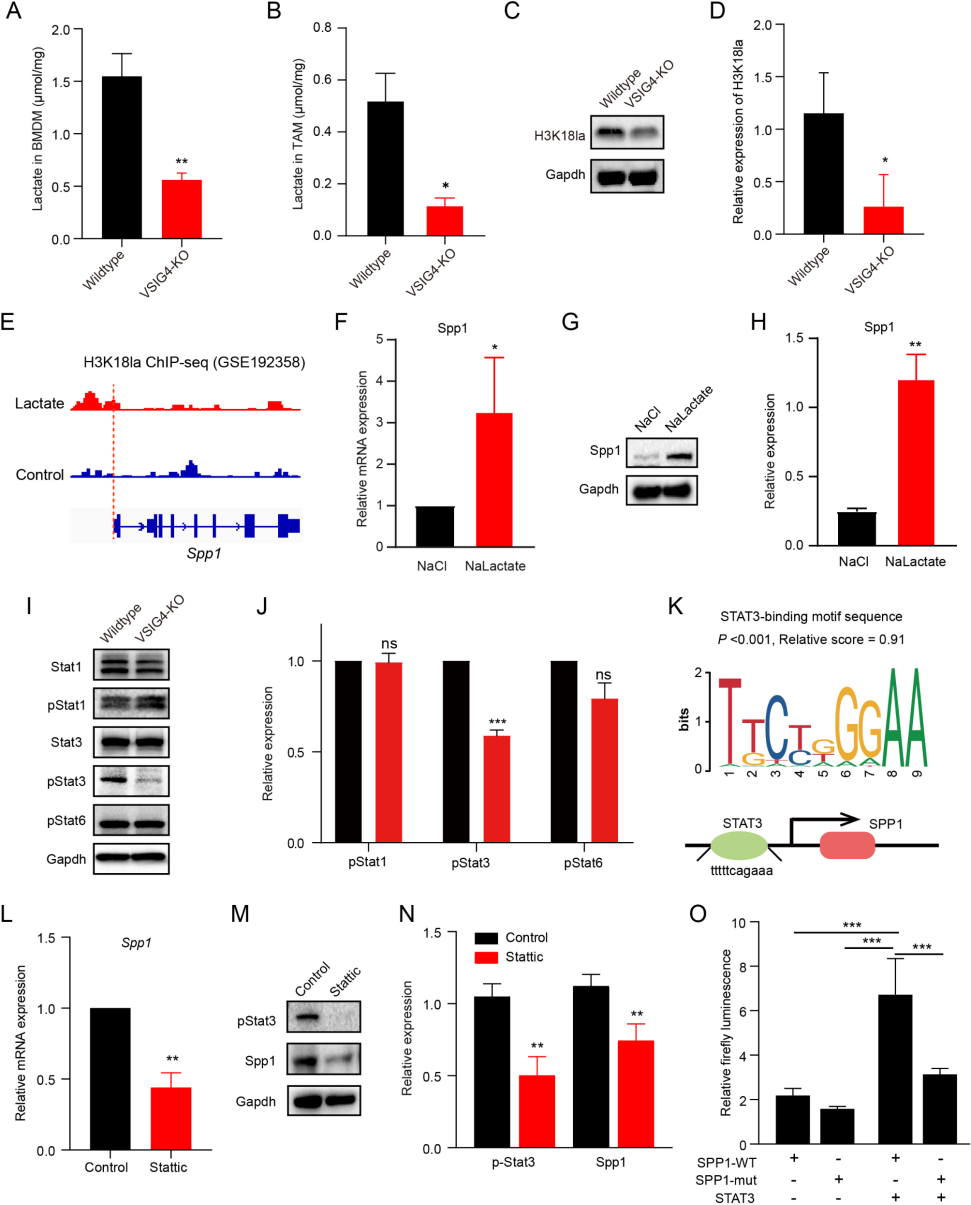
VSIG4 promoted SPP1 expression through enhancing the histone H3 lysine 18 lactylation and promoting the transcription activity of STAT3. (**A**) Co-culture of mATC with BMDMs isolated from VSIG4-KO and WT mice to examine the lactate concentration. (**B**) Isolation of TAMs from mATC-derived tumors in VSIG4-KO (*n* = 3) and WT mice (*n* = 3) on day 32 to examine the lactate concentration. Data are presented as mean ± S.E.M. (**C**-**D**) Co-culture of mATC with BMDMs isolated from VSIG4-KO and WT mice to examine the expressions of histone H3 lysine 18 lactylation (H3K18la). (**E**) Genome browser track analysis using dataset GSE192358 showed the H3K18la lactylation levels in the binding region of *Spp1* with or without the lactate treatment, where the left area of the dotted line represented the binding region of H3K18la on the *Spp1* promotor. The mRNA (**F**) and protein (**G**-**H**) level of Spp1 in BMDM with or without the lactate treatment (*n* = 3). (**I**-**J**) The expression of Stat1, pStat1, Stat3, pStat3, and pStat6 in BMDMs after VSIG4 knockout (*n* = 3). (**K**) The JASPAR database predicted the presence of STAT3 binding sites in the SPP1 promoter region. The mRNA (**L**) and protein (**M**-**N**) level of Spp1 in BMDMs after STAT3 inhibitor Stattic treatment (*n* = 3). (**O**) The transcriptional activity of STAT3 on SPP1 was determined by dual luciferase reporter gene assay (*n* = 3). Data are presented as mean ± S.D. *P < 0.05, **P < 0.01, ***P < 0.001

Because SPP1 signaling in VSIG4-KO TAMs was significantly reduced, the mechanism was further explored. Lactate has been emerging in epigenetic regulation by its nonmetabolic role, and lactate-mediated histone lysine lactylation involved in macrophage transition [32]. Given that VSIG4 was previously reported participating in reprogramming pyruvate metabolism in inflammatory diseases, we sought to explore the effect of VSIG4 on lactate production and lactate-mediated histone lysine lactylation. The lactate production in BMDMs with VSIG4 deficiency was significantly decreased compared with that in wild-type BMDMs (Fig. 6A). Notably, similar results were also found in TAMs isolated from mATC-derived tumors, as TAMs in VSIG4-KO mice showed lower intracellular lactate concentration compared to those in wild-type mice (Fig. 6B). Moreover, histone H3 lysine 18 lactylation (H3K18la) was also evidently attenuated in VSIG4-knockout BMDMs (Fig. 6C-D). By analysis of ChIP-seq data in a public dataset GSE192358, an increase of H3K18la peaks was observed at the promotor of *Spp1* after lactate treatment (Fig. 6E). Of note, in vitro lactate treatment significantly promoted the mRNA and protein expression of Spp1 in BMDMs (Fig. 6F-H).

The STAT transcription factor has been reported to regulate SPP1 transcription [33, 34], we hypothesized that VSIG4 potentially regulates SPP1 expression through STAT transcription factors. The screen of STAT family members revealed that knocking out VSIG4 significantly inhibited the phosphorylation of STAT3, but not STAT1 or STAT6 (Fig. 6I-J). The JASPAR database also predicted the presence of STAT3 binding sites in the SPP1 promoter region (*P* < 0.001, score = 0.91) (Fig. 6K). BMDMs treated with STAT3 inhibitor Stattic showed significantly lower expression of Spp1 both at mRNA and protein level (Fig. 6L-N). Dual luciferase reporter gene assay confirmed that STAT3 directly activated the transcription of *SPP1*, while this transcriptional regulation lost when its binding site at the promotor of *SPP1* was mutated (Fig. 6O).

Taken together, these results suggesting VSIG4 epigenetically facilitated SPP1 expression by enhancing the lactate-mediated H3K18la and promoting the transcription activity of STAT3.

## Discussion

Tumor heterogeneity leads to intricate immune evasion mechanisms, among which the activation of various immune checkpoints poses a major challenge in PD-1/PD-L1 treatment [35, 36]. The highly heterogenous expression of PD-L1 in different tumors is a major obstacle in clinical treatment. Substantial efforts have been made to identify next-generation immune checkpoints and immunomodulatory strategies that are promising in patients refractory to PD-1 blockade [37–41]. VSIG4 is a well-recognized checkpoint dysregulated in different diseases, while its role and mechanism in TAM-mediated immune evasion in aggressive cancers remains elusive. Here, we revealed the functions and mechanism of VSIG4^+^ TAMs in mediating tumor immune evasion.

The dysfunction of VSIG4 is closely related to immune-mediated inflammatory diseases, aging, and cancer [12, 42]. Studies have revealed that VSIG4 is a poor prognostic factor in patients with high-grade glioma, gastric cancer, and ovarian cancer [11, 22, 43]. Our previous studies have identified VSIG4^+^ TAMs as an emerging immune subset frequently infiltrating ATC, and the fold change of VSIG4 ranked top among various immune checkpoints compared with normal tissues [21]. Although tumor growth in murine lung cancer was significantly suppressed in VSIG4-deficiency mice and VSIG4 blockade showed anti-tumor activity in fibrosarcoma [13, 44], the exact role and mechanism of VSIG4^+^ TAMs in aggressive cancers remain unclear. In the present study, we found that the deletion of VSIG4 effectively inhibited the development and metastasis of cancers, and the anti-VSIG4 antibody had potent therapeutic effects in a variety of mouse transplanted tumor models. Given that patients with ATC have a median overall survival of only 4.8 months, the profound anti-tumor effect of VSIG4 blockade provides a promising opportunity for immunotherapy. Although ATC frequently harbors *BRAF*^V600E^ mutation [45–47], the outcomes of BRAF inhibitors are unsatisfactory owing to the common development of resistance [30, 31]. Our previous study demonstrated infiltration of VSIG4^+^ TAMs was highly correlated with activity of BRAF and RAS signaling in ATC [22]. Notably, the combination of VSIG4 blockade and BRAF inhibitor remarkably improved the anti-tumor activity, which provides a translational opportunity for ATC treatment.

VSIG4 can mediate immune tolerance by inhibiting activation of CD4^+^ and CD8^+^ T cell and facilitating the differentiation of Tregs [48, 49]. Analysis of clinical tumor samples indicated that high expression of VSIG4 correlated with low CD8^+^ T cell infiltration and high Foxp3^+^/CD8^+^ T cell ratio [11]. However, little is known about the impact of VSIG4^+^ TAMs on the tumor immune microenvironment in vivo. A previous report showed that knockdown of VSIG4 in human pancreatic cancer cells attenuated tumor growth and metastasis in vivo, while the immune microenvironment was missed due to the disadvantage of the nude mouse xenograft model [50]. Therefore, identifying the mechanism underlying the anti-tumor immunity of VSIG4 inhibition would enhance the comprehension of VSIG4-based immunotherapy. Our study revealed that in VSIG4-deficiency mice bearing lymphoma, a model widely used in immunotherapy and tumor microenvironment study [51, 52], the frequency of tumor-infiltrating CD8^+^ T and NK cells was evidently increased, and neutrophils as well as Tregs were profoundly decreased. Similar trends were observed in mice with VSIG4 blockade. Notably, VSIG4 knockout recovered the expression of antigen-presentation genes (*H2-K1*, *B2m*, and *H2-D1*) in TAMs, and targeting VSIG4^+^ TAMs significantly promoted the tumor infiltration and proliferation of antigen-specific CD8^+^ T cells. Our previous study showed that CD8^+^ T cells were intensively surrounded by VSIG4^+^ TAMs [21], which supports the notion that VSIG4^+^ TAMs can impair antigen-specific immunity orchestrated by macrophage and CD8^+^ T.

TAMs are the most frequent immune subpopulation and are characterized by high plasticity in tumors. The reprogramming of TAMs from a pro-tumor status to an anti-tumor phenotype has attracted considerable attention [53, 54]. However, the influence of VSIG4 inhibition on the TAM phenotype in the tumor microenvironment is poorly understood. SPP1^+^ TAMs have recently been identified as an emerging subset involved in T cell suppression, tumor progression, and drug resistance [55–57]. The extensive crosstalk between TAMs and stromal and/or tumor cells by SPP1 and its receptors underscores their importance. Our results demonstrated that SPP1 was significantly decreased in TAMs from VSIG4-deficiency mice compared to those from wild-type mice, which was also confirmed in vitro by co-culture assay. Notably, cell-cell interaction analysis revealed that SPP1 signaling between TAMs and tumor cells and/or neutrophils dramatically decreased in TAMs from VSIG4-deficiency mice. The proportion of immune cells showed that neutrophils and exhausted CD4^+^ T cells profoundly decreased in the VSIG4-KO group. Given that neutrophils have been reported to be the key cell subpopulation that inhibits T cell functions [58–60], our results extend the notion that VSIG4-mediated SPP1 signaling is essential for shaping the immunosuppressive microenvironment. We noticed that SPP1^+^ VSIG4^−^ TAMs also possessed strong activity of neutrophil chemotaxis. To overcome the heterogeneity of TAMs, VSIG4 and SPP1 were simultaneously inhibited and we found a synergic anti-tumor effect against ATC, highlighting targeting VSIG4-SPP1 axis as an emerging target for cancer immunotherapy.

Metabolic reprogramming affects macrophage polarization and function by metabolites, such as lactate [61, 62]. The utility of lactate induces M2 polarization of TAMs, which in turn promotes the aggressive phenotype of glioma cells [63]. In inflammatory diseases, VSIG4 activates the PI3K/Akt-STAT3 pathway to upregulate pyruvate dehydrogenase kinase-2, resulting the decrease of pyruvate oxidation and mitochondrial reactive oxygen species [18]. However, little is known about the role of VSIG4 in metabolic reprogramming of TAMs. Our study revealed VSIG4 knockout significantly reduced intracellular lactate production both in co-cultured BMDMs and TAMs isolated from ATC-derived tumors. More and more evidences support that lactate accumulation links to epigenetic regulation by its nonmetabolic role, which influences macrophage transition through lactate-mediated histone lysine lactylation [32]. Nonetheless, the relationship between lactate and VSIG4-mediated SPP1 expression remains poorly understood. In this study, we found VSIG4 facilitated lactate production and enhanced H3K18la, and treatment of lactate increased H3K18la accumulation at the promotor of SPP1, which promoted SPP1 expression. Study shows that occupancy of VSIG4 enabled the phosphorylation of MS4A6D, resulting in the activation of JAK2-STAT3-A20 signaling, which suppressed Nlrp3 and Il-1β expression [64]. Our study uncovered that VSIG4 promoted the phosphorylation of STAT3 and thereby the transcription activity of STAT3 on *SPP1*, conferring an oncogenic phenotype upon TAMs. Hence, VSIG4 epigenetically regulates SPP1 expression by enhancing lactate-mediated H3K18la accumulation and STAT3 activity, fostering an immunosuppressive phenotype of TAMs. Nonetheless, the process by which SPP1^+^ macrophages recruit tumor-associated neutrophils still require further research.

Taken together, our results demonstrate VSIG4-H3K18la-SPP1 signaling, beyond the checkpoint function of VSIG4 directly inhibiting T cells, confers on TAMs an emerging way to foster the immunosuppressive microenvironment and impair antigen-specific immunity against aggressive cancers.

## Electronic supplementary material

Below is the link to the electronic supplementary material.

**Supplementary Material 1**: **Table S1**: The antibody information. **Table S2**: The primer information. **Figure S1**: The expression of VSIG4 in murine pancreatic tumor. (A) The VSIG4 expression in BMDM and BMDC derived from bone marrow of wild-type mice. (B-C) The proportion and mean fluorescent intensity of VSIG4 in tumor cells, macrophages, MDSCs, and dendritic cells were determined by flow cytometry in Panc02-derived tumors (n = 4). Tumors were isolated on day 21. **Figure S2**: The therapeutic effect of VSIG4 blockade in mouse lymphoma, melanoma and ATC animal models. (A-C) The tumor growth curve, tumor weight and body weight of control, VSIG4-KO or VSIG4-KO combined with anti-VSIG4 group in lymphoma tumor model implanted with E.G7-OVA. (D-E) The tumor growth curve and weight of control, VSIG4-KO or VSIG4-KO combined with anti-VSIG4 group in melanoma tumor models subcutaneously injected with B16F10. (F-H) The mATC tumor-bearing mice were treated with anti-VSIG4 and/or PLX4720 as single agent and combination therapy, respectively. The tumor growth curve, body weight, and tumor weight of each group were recorded. Data are presented as mean ± S.E.M. *P < 0.05, **P < 0.01, ***P < 0.001. **Figure S3**: Targeting VSIG4 enhanced anti-tumor immune microenvironment. (A) Diagram of the multicolor flow immunophenotype panel to analyze the immune cells. (B-C) The splenic proportions of lymphocytes in pancreatic tumor-bearing mice were examined by flow cytometry IgG isotype and anti-VSIG4 groups (n = 6). (D-H) The proportions of tumor infiltrating lymphocytes (TILs) in ATC tumor-bearing mice were measured by flow cytometry after anti-VSIG4 treatment (n = 6). Data are presented as mean ± S.E.M. *P < 0.05, **P < 0.01. **Figure S4**: Infiltration of different myeloid ant T cell subsets in mATC-derived tumors after VSIG4 knockout. (A) The single-cell transcriptome analysis of the composition of microenvironmental cells. Samples were isolated from mATC-derived tumors in VSIG4-KO and WT mice. (B-C) The UMAP map and cell markers of different subtypes of T lymphocytes. (D-E) The single-cell transcriptome analysis of the composition of myeloid cells after VSIG4 knockout. (F) The expression of neutrophil markers in different myeloid cell subsets. (G) The UMAP map of wildtype and VSIG4-KO macrophages. **Figure S5**: The differences of ligand-receptor communication after VSIG4 knockout. (A) The differences of ligand-receptors in macrophage communicated with other cells after VSIG4 knockout. (B) The differences of CCL6-CCR1/2 and CSF signaling in cell communication after VSIG4 knockout.

## Data Availability

All data needed to evaluate the conclusions are included in this article and/or in its supplemental material. The data within the study are available upon request from the corresponding author.
