# Supplementary material for: VSIG4+ tumor-associated macrophages mediate neutrophil infiltration and impair antigen-specific immunity in aggressive cancers through epigenetic regulation of SPP1
Source: J Exp Clin Cancer Res. 2025 Feb 7;44:45. doi: 10.1186/s13046-025-03303-z (PMC11803937; doi:10.1186/s13046-025-03303-z)
Supplement: Supplementary file 1 — Supplementary Material 1: Table S1: The antibody information. Table S2: The primer information. Figure S1: The expression of VSIG4 in murine pancreatic tumor. (A) The VSIG4 expression in BMDM and BMDC derived from bone marrow of wild-type mice. (B-C) The proportion and mean fluorescent intensity of VSIG4 in tumor cells, macrophages, MDSCs, and dendritic cells were determined by flow cytometry in Panc02-derived tumors (n = 4). Tumors were isolated on day 21. Figure S2: The therapeutic effect of VSIG4 blockade in mouse lymphoma, melanoma and ATC animal models. (A-C) The tumor growth curve, tumor weight and body weight of control, VSIG4-KO or VSIG4-KO combined with anti-VSIG4 group in lymphoma tumor model implanted with E.G7-OVA. (D-E) The tumor growth curve and weight of control, VSIG4-KO or VSIG4-KO combined with anti-VSIG4 group in melanoma tumor models subcutaneously injected with B16F10. (F-H) The mATC tumor-bearing mice were treated with anti-VSIG4 and/or PLX4720 as single agent and combination therapy, respectively. The tumor growth curve, body weight, and tumor weight of each group were recorded. Data are presented as mean ± S.E.M. *P < 0.05, **P < 0.01, ***P < 0.001. Figure S3: Targeting VSIG4 enhanced anti-tumor immune microenvironment. (A) Diagram of the multicolor flow immunophenotype panel to analyze the immune cells. (B-C) The splenic proportions of lymphocytes in pancreatic tumor-bearing mice were examined by flow cytometry IgG isotype and anti-VSIG4 groups (n = 6). (D-H) The proportions of tumor infiltrating lymphocytes (TILs) in ATC tumor-bearing mice were measured by flow cytometry after anti-VSIG4 treatment (n = 6). Data are presented as mean ± S.E.M. *P < 0.05, **P < 0.01. Figure S4: Infiltration of different myeloid ant T cell subsets in mATC-derived tumors after VSIG4 knockout. (A) The single-cell transcriptome analysis of the composition of microenvironmental cells. Samples were isolated from mATC-derived tumors in VSIG4-KO and WT mice. (B-C) The [file 13046_2025_3303_MOESM1_ESM.doc]

**Supplemental material**

**MATERIALS AND METHODS**

**Table S1. The antibody information.**

| **Antibodies** | **Source** | **Clone** | **Identifier** |
| --- | --- | --- | --- |
| Anti-Mouse CD4 | BioLegend | GK1.5 | Cat#：100434 |
| Anti-Mouse CD8a | BioLegend | 53-6.7 | Cat#：100752 |
| Anti-Mouse Gr-1 | BioLegend | RB6-8C5 | Cat#：108442 |
| Anti-Mouse CD11b | BioLegend | M1/70 | Cat#：101206 |
| Anti-Mo/Rt FOXP3 | Invitrogen | FJK-16s | Cat#：2430489 |
| Anti-Mouse CD3 | BD Horizon™ | 17A2 | Cat#：564010 |
| Anti-Mouse NK-1,1 | BD Pharmingen™ | PK136 | Cat#：557391 |
| Anti-Mouse CD25 | BD Pharmingen™ | PC61 | Cat#：557192 |
| Anti-Mouse CD45 | BD Pharmingen™ | 30-F11 | Cat#：557659 |
| Anti-Mouse F4/80 | BioLegend | BM8 | Cat#：123110 |
| Anti-Mouse CD163 | BioLegend | S15049I | Cat#：155320 |
| Anti-Mouse CD11c | BD Pharmingen™ | HL3 | Cat#：550261 |
| Anti-H2Kb-OVA_257-264_ Antibody | BioLegend | 25-D1.16 | Cat#：141608 |
| Anti-mouse/human CD11b | BioLegend | M1/70 | Cat#：101216 |
| Anti-mouse/human CD11b | BioLegend | M1/70 | Cat#：101206 |
| Anti-Mouse F4/80 | BioLegend | BM8 | Cat#：123114 |
| Anti-Mouse Ly-6G | BioLegend | 1A8 | Cat#：127645 |
| Anti-Mouse CD8a | BD Pharmingen™ | 53-6.7 | Cat#：553030 |
| Anti-Mouse CD3 | BioLegend | 17A2 | Cat#：100232 |

**Table S2. The primer information.**

| **Genes** | **Primer（5' to 3'）** |
| --- | --- |
| H2-d1-F | TGGTGCTGCAGAGCATTACA |
| H2-d1-R | TGTGCCTTTGGGGAATCTGT |
| H2-k1-F | GAAAGCCAAGGGCAATGAGC |
| H2-k1-R | TGAATAGTGTGAGAGCCGCC |
| B2m-F | TCACACTGAATTCACCCCCA |
| B2m-R | TCACATGTCTCGATCCCAGT |
| Spp1-F | ATCTCACCATTCGGATGAGTCT |
| Spp1-R | TGTAGGGACGATTGGAGTGAAA |
| Gapdh-F | GCCTTCCGTGTTCCTACC |
| Gapdh-R | GCCTGCTTCACCACCTTC |

**RESULTS**


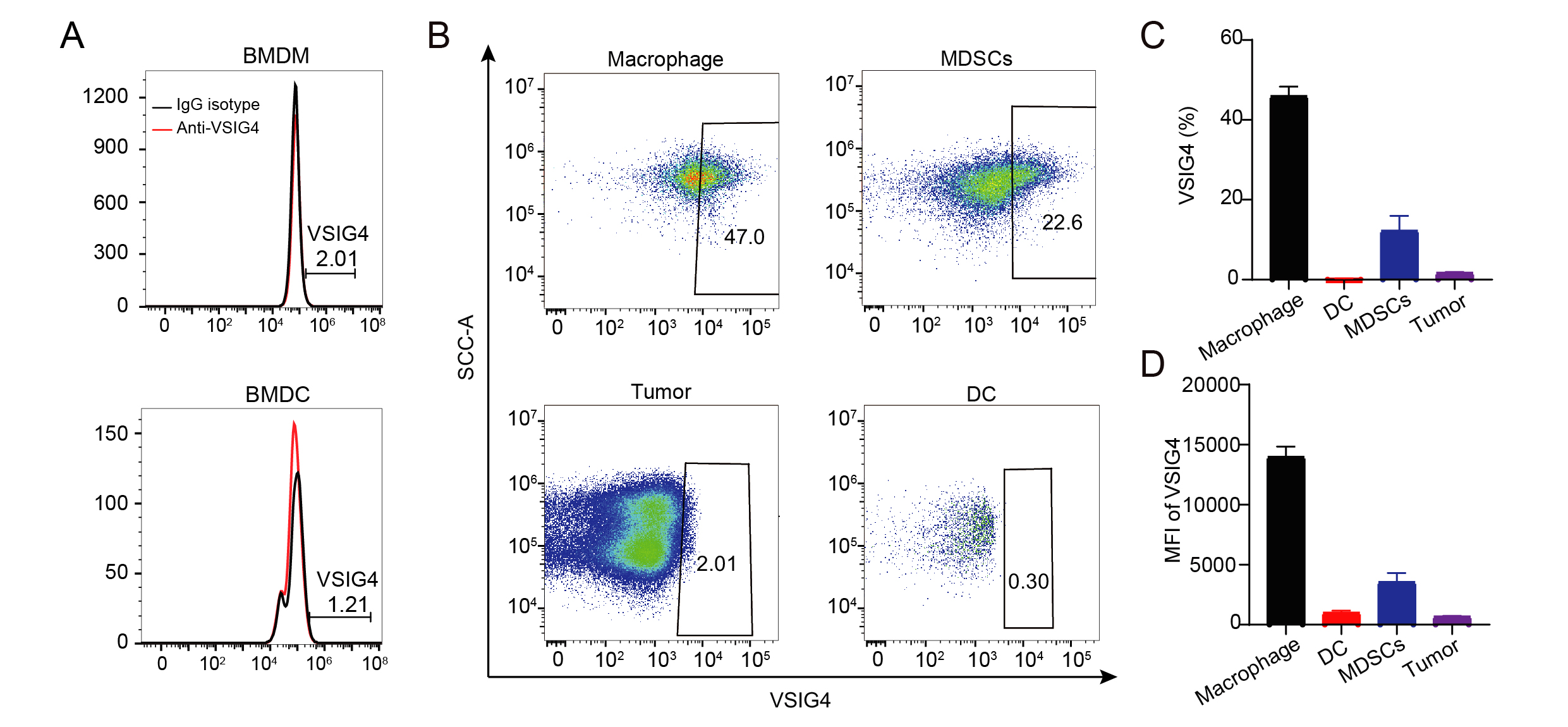


**Figure S1. The expression of VSIG4 in murine pancreatic tumor.** (A) The VSIG4 expression in BMDM and BMDC derived from bone marrow of wild-type mice. (B-C) The proportion and mean fluorescent intensity of VSIG4 in tumor cells, macrophages, MDSCs, and dendritic cells were determined by flow cytometry in Panc02-derived tumors (n = 4). Tumors were isolated on day 21.


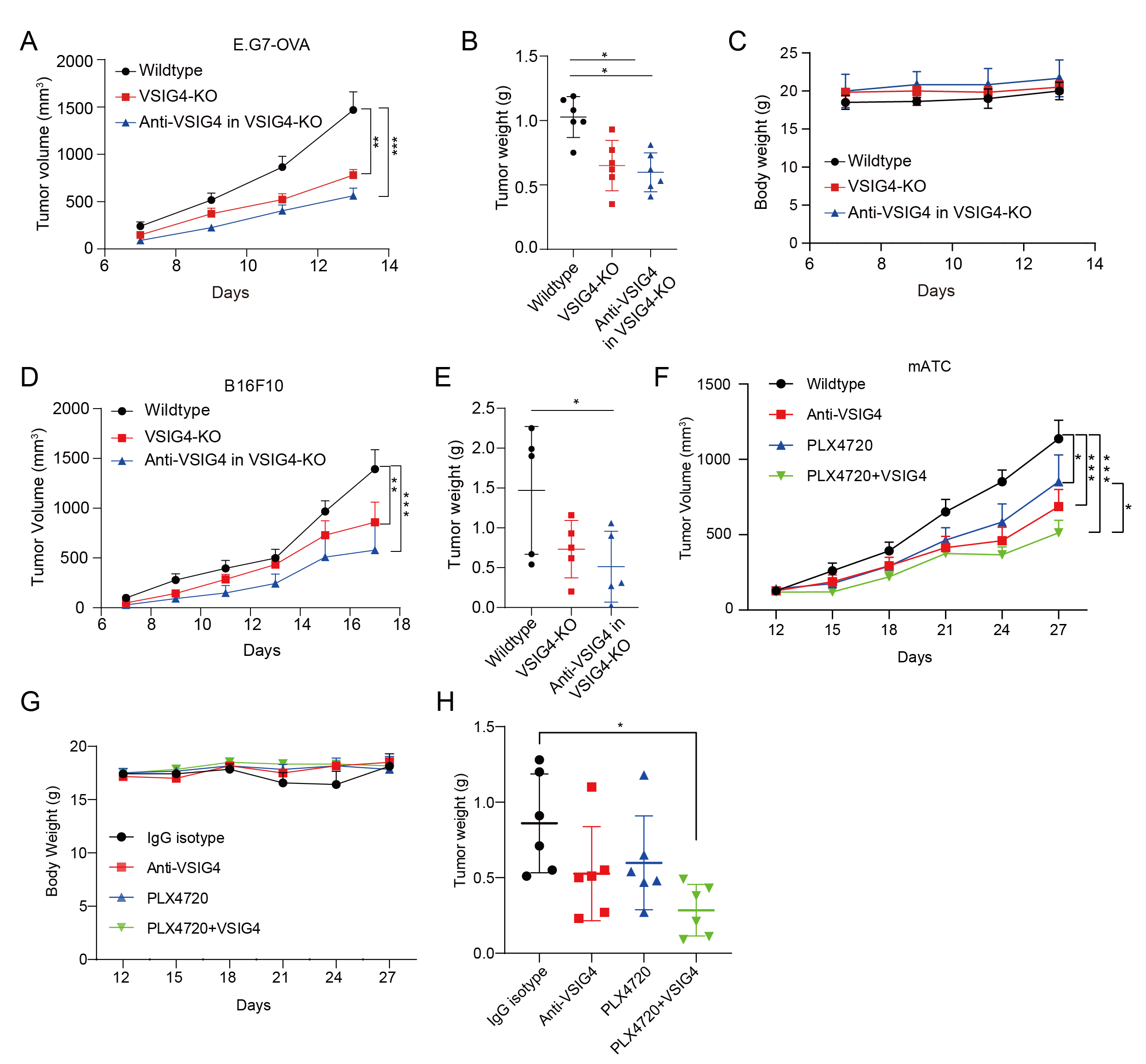


**Figure S2. The therapeutic effect of VSIG4 blockade in mouse lymphoma, melanoma and ATC animal models.** (A-C) The tumor growth curve, tumor weight and body weight of control, VSIG4-KO or VSIG4-KO combined with anti-VSIG4 group in lymphoma tumor model implanted with E.G7-OVA. (D-E) The tumor growth curve and weight of control, VSIG4-KO or VSIG4-KO combined with anti-VSIG4 group in melanoma tumor models subcutaneously injected with B16F10. (F-H) The mATC tumor-bearing mice were treated with anti-VSIG4 and/or PLX4720 as single agent and combination therapy, respectively. The tumor growth curve, body weight, and tumor weight of each group were recorded. Data are presented as mean ± S.E.M. **P* < 0.05, ***P* < 0.01, ****P* < 0.001.


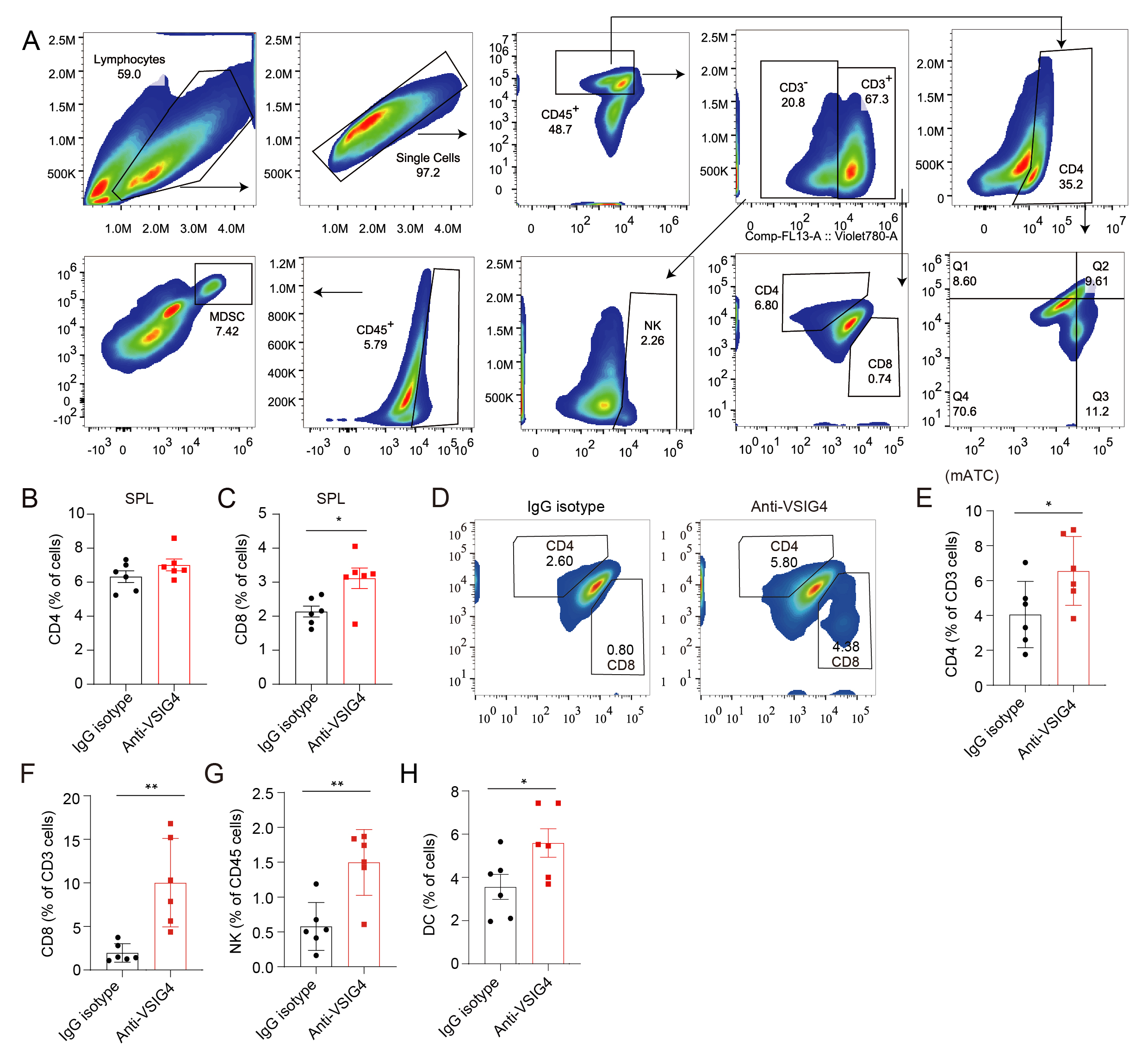


**Figure S3. Targeting VSIG4 enhanced anti-tumor immune microenvironment.** (A) Diagram of the multicolor flow immunophenotype panel to analyze the immune cells. (B-C) The splenic proportions of lymphocytes in pancreatic tumor-bearing mice were examined by flow cytometry IgG isotype and anti-VSIG4 groups (n = 6). (D-H) The proportions of tumor infiltrating lymphocytes (TILs) in ATC tumor-bearing mice were measured by flow cytometry after anti-VSIG4 treatment (n = 6). Data are presented as mean ± S.E.M. **P* < 0.05, ***P* < 0.01.


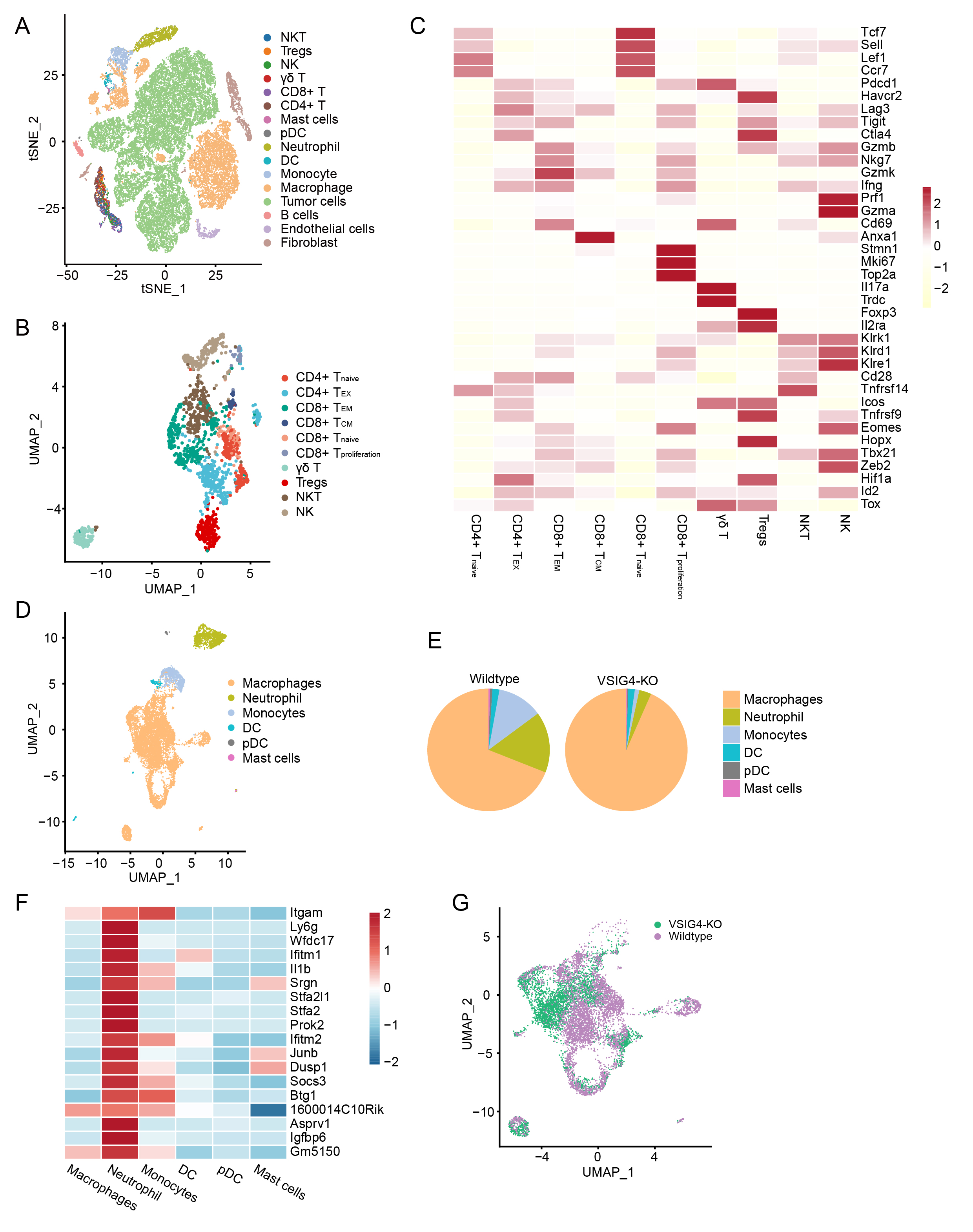


**Figure S4. Infiltration of different myeloid ant T cell subsets in mATC-derived tumors after VSIG4 knockout.** (A) The single-cell transcriptome analysis of the composition of microenvironmental cells. Samples were isolated from mATC-derived tumors in VSIG4-KO and WT mice. (B-C) The UMAP map and cell markers of different subtypes of T lymphocytes. (D-E) The single-cell transcriptome analysis of the composition of myeloid cells after VSIG4 knockout. (F) The expression of neutrophil markers in different myeloid cell subsets. (G) The UMAP map of wildtype and VSIG4-KO macrophages.


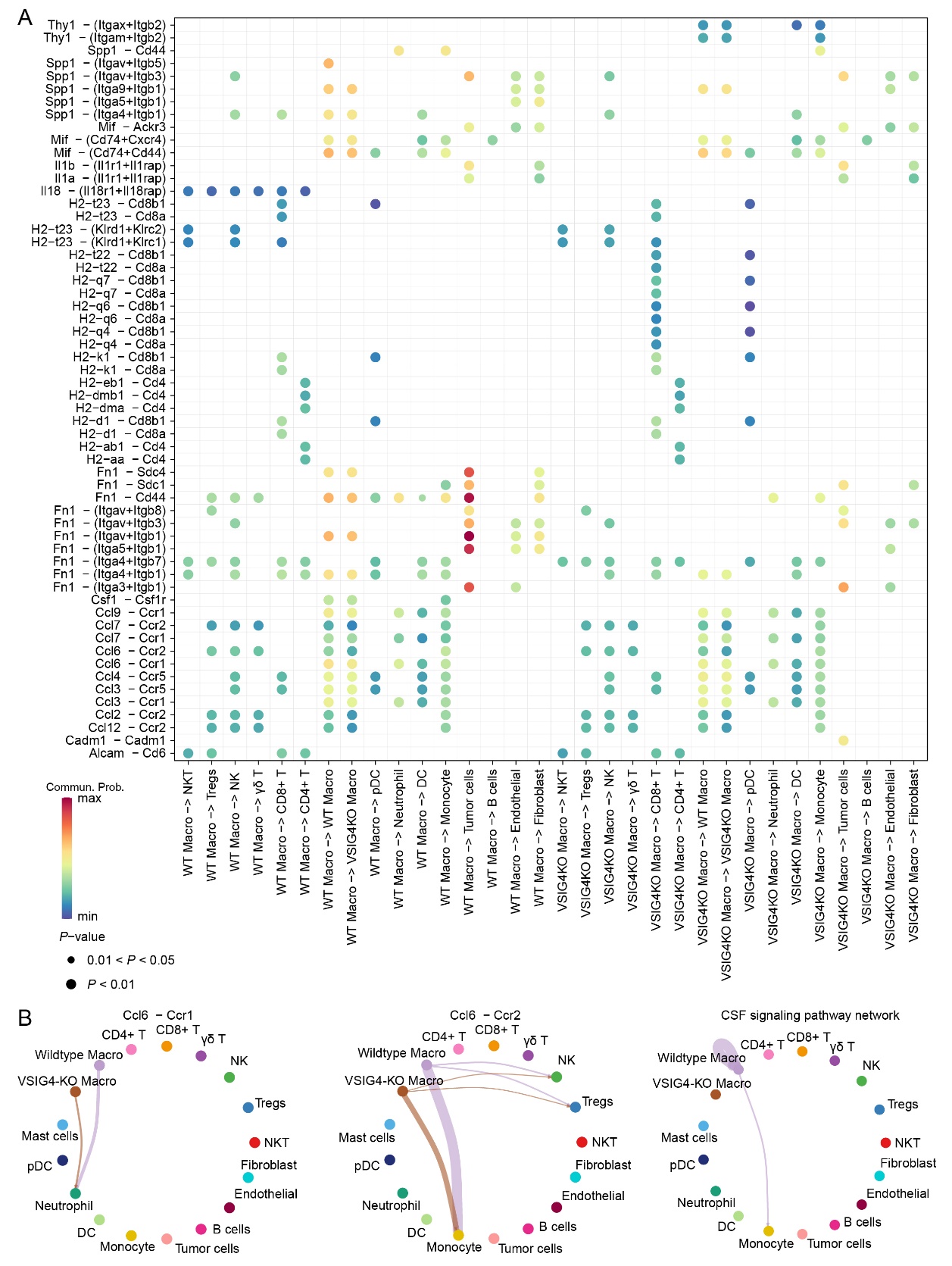


**Figure S5. The differences of ligand-receptor communication after VSIG4 knockout.** (A) The differences of ligand-receptors in macrophage communicated with other cells after VSIG4 knockout. (B) The differences of CCL6-CCR1/2 and CSF signaling in cell communication after VSIG4 knockout.
